# Two mechanisms drive pronuclear migration in mouse zygotes

**DOI:** 10.1038/s41467-021-21020-x

**Published:** 2021-02-05

**Authors:** Kathleen Scheffler, Julia Uraji, Ida Jentoft, Tommaso Cavazza, Eike Mönnich, Binyam Mogessie, Melina Schuh

**Affiliations:** 1 Department of Meiosis, Max Planck Institute for Biophysical Chemistry, Göttingen, Germany; 2 School of Biochemistry, University of Bristol, Bristol, UK

**Keywords:** Cytoskeleton, Embryogenesis

## Abstract

A new life begins with the unification of the maternal and paternal chromosomes upon fertilization. The parental chromosomes first become enclosed in two separate pronuclei near the surface of the fertilized egg. The mechanisms that then move the pronuclei inwards for their unification are only poorly understood in mammals. Here, we report two mechanisms that act in concert to unite the parental genomes in fertilized mouse eggs. The male pronucleus assembles within the fertilization cone and is rapidly moved inwards by the flattening cone. Rab11a recruits the actin nucleation factors Spire and Formin-2 into the fertilization cone, where they locally nucleate actin and further accelerate the pronucleus inwards. In parallel, a dynamic network of microtubules assembles that slowly moves the male and female pronuclei towards the cell centre in a dynein-dependent manner. Both mechanisms are partially redundant and act in concert to unite the parental pronuclei in the zygote’s centre.

## Abstract (web summary)

In a newly fertilized egg, maternal and paternal chromosomes are enclosed in two separate pronuclei but the mechanisms in mammals for pronuclear movement are unclear. Here, the authors report that both F-actin and microtubule polymerization act in concern to drive inward movement of pronuclei towards the cell centre.

## Introduction

Fertilization unites the father’s and the mother’s chromosomes to create a genetically unique embryo. Following sperm entry, the egg completes the second meiotic division and the male and female pronuclei subsequently form in proximity of the zygote’s surface^1–3^, which is lined by an actin cortex. From the zygote’s periphery, they need to move inwards in order to unite the paternal and maternal chromosomes on the first mitotic spindle. Recent studies revealed that incomplete pronuclear migration leads to defects during embryo development in mammals. If the pronuclei are too far apart when they break down, two entirely separate spindles will form^4,5^. Zygotes with two separate spindles are more likely to form multiple nuclei upon the first mitotic division, and often fail to develop into healthy embryos^4,6^.

Despite the importance of pronuclear migration, the mechanisms that unite male and female pronuclei in mammals are poorly understood. Work that was mostly carried out in non-mammalian species has established essential functions for microtubules and dynein in pronuclear migration^7–16^. Dynein anchored in the cytoplasm is thought to move the male pronucleus inwards by pulling on the sperm aster that is associated with the male pronucleus^15,17,18^. The female pronucleus recruits dynein on its nuclear envelope and moves towards the male pronucleus along the sperm aster^19^. Pronuclear centration is further promoted by pushing of pronucleus-associated microtubules against the zygote’s cortex^8,20^. Microtubule-dependent pushing similarly drives nuclear movements in *Drosophila* oocytes^21^ and fission yeast^22^.

Although mouse zygotes contain a dense microtubule network and acentriolar microtubule-organizing centres (aMTOCs)^23,24^, pronuclear migration in this system is thought to rely more on the actin cytoskeleton^5,25–28^. How actin helps to centre pronuclei before cell division is unclear.

Here, we developed an experimental system that allowed us to follow and experimentally probe the entire process of pronuclear migration in live mouse zygotes. By combining it with loss-of-function assays, we identified two distinct mechanisms that drive pronuclear migration. In the first mechanism, the male pronucleus is initially moved inwards by the flattening fertilization cone. At the same time, the actin nucleation factors Formin-2 and Spire are recruited into the fertilization cone in a Rab11a-dependent manner, where they locally nucleate actin, accelerating the pronucleus’ inwards movement and detaching it from the plasma membrane. In the second mechanism, which acts throughout the central region of the zygote, a cytoplasmic microtubule network forms and carries the pronuclei inwards in a dynein-dependent manner. Both mechanisms act in concert and are partially redundant, which increases the likelihood of successful pronuclear migration. Thus, both the actin and microtubule cytoskeletons actively participate in the unification of parental chromosomes in mouse zygotes.

## Results

### Pronuclear migration is driven by distinct mechanisms in the periphery and in the central region of the zygote

The formation of mammalian pronuclei and the early stages of their migration have not been visualized previously. To observe early events in live zygotes immediately after fertilization (Supplementary Fig. 1a), we microinjected mRNAs encoding fluorescent markers into eggs before in vitro fertilization (IVF), by adapting a particularly gentle and quantitative method for microinjection^29^. We found this approach is compatible with high fertilization rates and mouse embryo developmental capacity (Supplementary Fig. 11a, b).

**Fig. 1:**
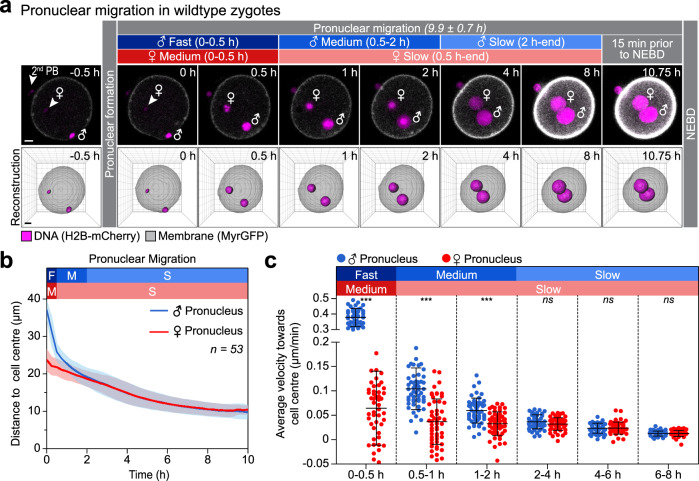
Pronuclei migrate by different modes in the periphery and central region of the zygote. **a** Three-dimensional time-lapse images (upper panel) and iso-surface reconstruction (lower panel) of pronuclei (H2B-mCherry, z-projection of 28 sections, every 3 µm) and the cell surface (MyrGFP, equatorial section) in live zygotes relative to pronuclear formation (0 h). Female (♀) and male (♂) genomes are labelled; as well as the second polar body (PB), which forms around excess maternal DNA in meiosis II. Distinct phases of migration for male and female pronuclei were defined according to migratory behaviour of male (blue) or female (red) pronuclei (for details see text), which were further characterized by typical speeds within certain ranges as follows: fast (>0.1 µm/min), medium (0.05–0.1 µm/min) and slow (<0.05 µm). Duration of pronuclear migration from pronuclear formation to nuclear envelope breakdown (NEBD) was quantified in Supplementary Fig. 1m. Scale bar, 10 μm. **b** The mean distance (thick line) of male (♂, blue) and female (♀, red) pronuclei centroids to zygote centre during pronuclear migration was calculated from data sets as shown in (**a**). Movement speeds are classified as in (**a**). Total number of analyzed zygotes specified in italics was pooled from five independent experiments. S.d. shown as shaded areas. **c** Statistical plots of average velocities of male (♂, blue) and female (♀, red) pronuclei during pronuclear migration calculated from data sets shown in (**b**) with the sample size as in (**b**). Statistical plots show mean (horizontal middle lines), and standard deviation (whiskers). Two-tailed Student’s *t* test was used to test for significance (from left to right: *p* < 0.0001, *p* < 0.0001, *p* < 0.0001, *p* = 0.09, *p* = 0.9 and *p* = 0.9).

Introduction of the markers histone H2B-mCherry and MyrGFP (myristoylated EGFP) into unfertilized eggs allowed us to, respectively, visualize chromatin and the cell surface in zygotes (Fig. 1a). In time-lapse microscopy experiments that captured images from early pronuclear formation to the first mitotic division of the mouse embryo, we recorded high-resolution z-stacks covering the entire zygote. We then reconstructed from fluorescence signals the pronuclei and cell volume in 3D to quantitatively assess pronuclear migration (Fig. 1a, b, Supplementary Fig. 1b, c and Supplementary Movie 1). The distances of the pronuclear centroids to the cell centre were calculated in 3D and then plotted over time (Fig. 1a, b and Supplementary Fig. 1d).

The male pronucleus formed directly underneath the cell surface, within the fertilization cone, at an average distance of 37.2 ± 2.3 µm from the cell centre (Fig. 1a and Supplementary Fig. 1a, e). During the first 30 min, it moved rapidly inwards with a mean velocity of 0.38 ± 0.06 µm/min (Fig. 1b, c). In the subsequent 30 min, its velocity decreased to 0.10 ± 0.04 µm/min. Over the following 7 h, its velocity decreased further, from ~0.04 to 0.01 µm/min (Fig. 1b, c). In contrast, the female pronucleus formed further inwards than the male pronucleus, at an average distance of 23.7 ± 2.7 µm from the cell centre (Fig. 1a and Supplementary Fig. 1a, e). During the first 30 min upon its formation, the female pronucleus moved with an average velocity of 0.06 ± 0.08 µm/min and was hence around six times slower than the male pronucleus (Fig. 1b, c). Over the following 7.5 h, its speed decreased further from ~0.04 to 0.01 µm/min. From 2 to 8 h upon pronuclear formation, the male and the female pronuclei moved at similar speeds, ranging between ~0.04 and 0.01 µm/min (Fig. 1c).

We repeated this analysis also in the outbred CD-1 mouse strain with similar results, suggesting that this movement pattern is conserved between strains (Supplementary Fig. 1f, g).

**Fig. 2:**
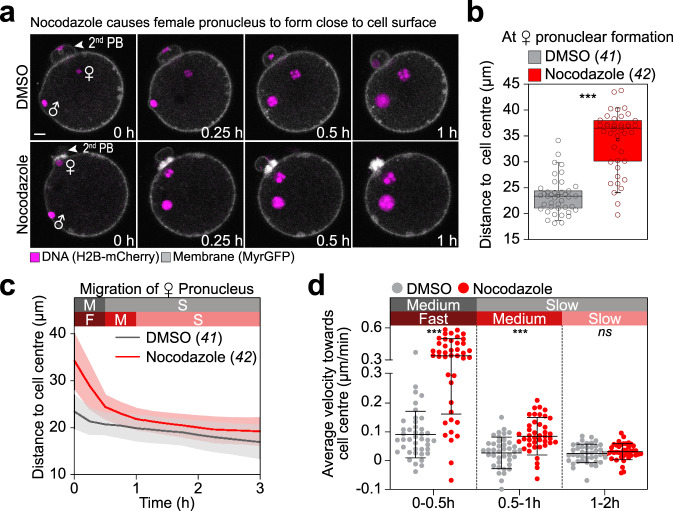
Female pronuclei have a pronounced fast migration phase when forced to form close to the cell surface. **a** Three-dimensional time-lapse images of pronuclei (H2B-mCherry) and the cell surface (MyrGFP) relative to pronuclear formation (0 h) in zygotes treated with DMSO or 1 µM nocodazole. Female (♀) and male (♂) genomes are labelled; as well as the second polar body (PB). Scale bar, 10 μm. **b** Distance of female pronuclei formation site from the cell centre relative to pronuclear formation (0 h) in DMSO- and nocodazole-treated zygotes calculated from data sets as shown in (**a**). All individual values (circles) are overlaid with a box plot showing median (line), mean (small square), 5th, 95th (whiskers) and 25th and 75th percentile (boxes enclosing 50% of the data) calculated from the total number of zygotes (italics) and pooled from four independent experiments. Two-tailed Student’s *t* test was used to test for significance (*p* < 0.0001). **c** The mean distance (thick line) of female (♀) pronuclei centroids to zygote centre relative to pronuclear formation (0 h) in DMSO- and nocodazole-treated zygotes was calculated from (**a**). Phases of respective movement speeds are classified as in Fig. 1a. Total number of analyzed zygotes (italics) was pooled from four independent experiments. S.d. shown as shaded areas. **d** Statistical plots of average velocities of female (♀) pronuclei during pronuclear migration in DMSO- or nocodazole-treated zygotes calculated from (**c**) with the same sample size as in (**c**). Statistical plots as in Fig. 1c. Two-tailed Student’s *t* test was used to test for significance (from left to right: *p* < 0.0001, *p* < 0.0001 and *p* = 0.3).

The male pronucleus was around six times faster during the initial phase of migration than the female pronucleus. We asked if this disparity is due to the different distances from the cell surface at which the male and female pronuclei were forming. To test this hypothesis, we forced the female pronucleus to form more peripherally (at an average distance of 34.2 ± 5.8 µm from the cell centre) (Fig. 2a, b and Supplementary Movie 2) by partially depolymerizing the spindle remnant that held the female pronucleus away from the surface (Supplementary Fig. 1h). Strikingly, the female pronucleus now showed a similar migratory pattern to the male pronucleus: during the first 30 min, it moved rapidly at an average speed of 0.33 ± 0.17 µm/min before adopting a slower speed, ranging between ~0.08 and 0.03 µm/min from 0.5 to 2 h (Fig. 2c, d). Conversely, in untreated zygotes, male pronuclei occasionally (3/56 zygotes; green dots in Supplementary Fig. 1e) formed at a distance from the cell surface, and then did not display the fast phase of migration (green curves in Supplementary Fig. 1i).

Together, these data establish that pronuclei move distinctly in different regions of the zygote. They adopt a rapid migration mode near the surface, and a slow migration mode throughout the centre. Male pronuclei normally form close to the surface and show both the first fast phase of migration as well as the second slower phase of migration. Female pronuclei typically form deeper inside the zygote and show a less pronounced fast migration phase, in addition to the second slower phase.

### Pronuclei migrate inwards in the absence of Rab11a-positive vesicles

What are the mechanisms that drive pronuclear migration in the periphery and in the central region? Nuclei also become centred in unfertilized, prophase-arrested oocytes^30^ where Rab11a-positive vesicles move via Myosin-5b motors in the cytoplasm^31^ and participate in nuclear movement^30,31^ by generating a pressure gradient^30^. A similar vesicle-dependent gradient could also be involved pronuclear centration in zygotes^5^.

**Fig. 3:**
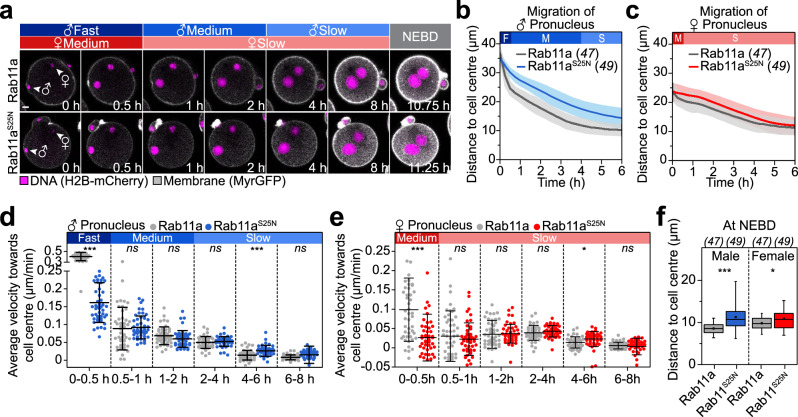
Rab11a is required for pronuclear migration in the periphery of the zygote. **a** Three-dimensional time-lapse images of pronuclei (H2B-mCherry) and the cell surface (MyrGFP) in zygotes expressing SNAP-Rab11a or SNAP-Rab11a^S25N^ until nuclear envelope breakdown (NEBD). Female (♀) and male (♂) genomes are shown at pronuclear formation (0 h). Phases with respective pronuclear movement speeds are classified as in Fig. 1a. Scale bar, 10 μm. The mean distance (thick line) of male (♂, (**b**)) and female (♀, (**c**)) pronuclei centroids to zygote centre during pronuclear migration in zygotes expressing SNAP-Rab11a or SNAP-Rab11a^S25N^ was calculated from data sets as shown in (**a**). Total number of analyzed zygotes specified in italics was pooled from six independent experiments. S.d. shown as shaded areas. Statistical plots of average velocities of male (♂, (**d**)) and female (♀, (**e**)) pronuclei during pronuclear migration in zygotes expressing SNAP-Rab11a or SNAP-Rab11a^S25N^ calculated from (**b**) and (**c**) with the same sample size as in (**b**) and (**c**), respectively. Statistical plots show mean ± s.d. Two-tailed Student’s *t* test was used to test for significance (from left to right: **d** *p* < 0.0001, *p* = 0.7, *p* = 0.08, *p* = 0.4, *p* < 0.0001 and *p* = 0.06; **e** *p* < 0.0001, *p* = 0.5, *p* = 0.8, *p* = 0.2, *p* = 0.02 and *p* = 0.5). We noticed that dominant-negative Rab11a strongly but not completely abolished the fast migration phase of male pronuclei (**d**). Detailed analysis of these early processes suggests that male pronuclei maintain contact to the plasma membrane for twice as long in dominant-negative Rab11a^S25N^ expressing zygotes (Supplementary Fig. 4j, l). Rapid nuclear expansion during this time-period (Supplementary Fig. S1k) together with a mildly delayed flattening of the cone (Supplementary Fig. 4k) may explain why fast migration may not be fully blocked upon inhibition of Rab11a. Furthermore, we noticed that pronuclei are slightly accelerated in Rab11a-overexpressing zygotes, which means that they move mildly faster over a longer period of time (Fig. S12p, q). **f** Distance of pronuclei centroids to zygote centre at NEBD in zygotes expressing SNAP-Rab11a or SNAP-Rab11a^S25N^ calculated from data sets in (**a**). Box plot showing median (line), mean (small square), 5th, 95th (whiskers) and 25th and 75th percentile (box) was calculated from the total number of analyzed zygotes specified in italics. Two-tailed Student’s *t* test was used to test for significance (male *p* < 0.0001 and female *p* = 0.03).

Live and fixed cell analysis showed that Rab11a-positive vesicles were present in early stages of pronuclear migration, but largely depleted during the late stages of pronuclear migration (Supplementary Fig. 2a–f). To test if Rab11a is important for pronuclear migration, we expressed a dominant-negative variant of Rab11a, or treated zygotes with brefeldin A (BFA)^32^, which depleted the vesicles in the zygote (Supplementary Fig. 2g–j). Interestingly, the male and female pronuclei still moved inwards under both conditions, demonstrating that Rab11a-positive vesicles are not essential for pronuclear migration (Fig. 3a–c, Supplementary Movie 3 and Supplementary Fig. 3a–c). Dominant-negative Rab11a and BFA decreased the velocity of pronuclear migration during the initial fast migratory phase, but not during the second slower phase (Fig. 3d, e and Supplementary Fig. 3d e). Moreover, pronuclear distances to the cell centre at nuclear envelope breakdown (NEBD) were only mildly increased upon expression of the dominant-negative Rab11a^S25N^ variant (by 30% for the male pronucleus and 10% for the female pronucleus; Fig. 3f).

If a pressure gradient was the main driving force behind pronuclear migration it should also move oil droplets inwards that have a similar size as pronuclei^33^. However, oil droplets ranging in diameter from 9 to 24 µm (female pronuclei grow from ~7 to 21 μm; male pronuclei from ~10 to 24 μm during migration; Supplementary Fig. 1j, k) did not move inwards unlike pronuclei (Supplementary Fig. 3f, g). Only oil droplets that were in close proximity of a male pronucleus as it formed in the fertilization cone rapidly moved inwards right after pronuclear formation, but later remained at their position or only underwent local random movements (Supplementary Fig. 3h–j and Supplementary Movie 4).

Together, these data indicate that pronuclear migration is not driven by global pressure gradients in mouse zygotes. Consistent with our findings, a recent study using microsensors to monitor forces in the cytoplasm of the zygote did not observe global inwards-directed forces acting on polyethylene microspheres or nanodevices^34^.

### The flattening fertilization cone moves the male pronucleus inward and recruits Rab11a, Formin-2 and Spire

**Fig. 4:**
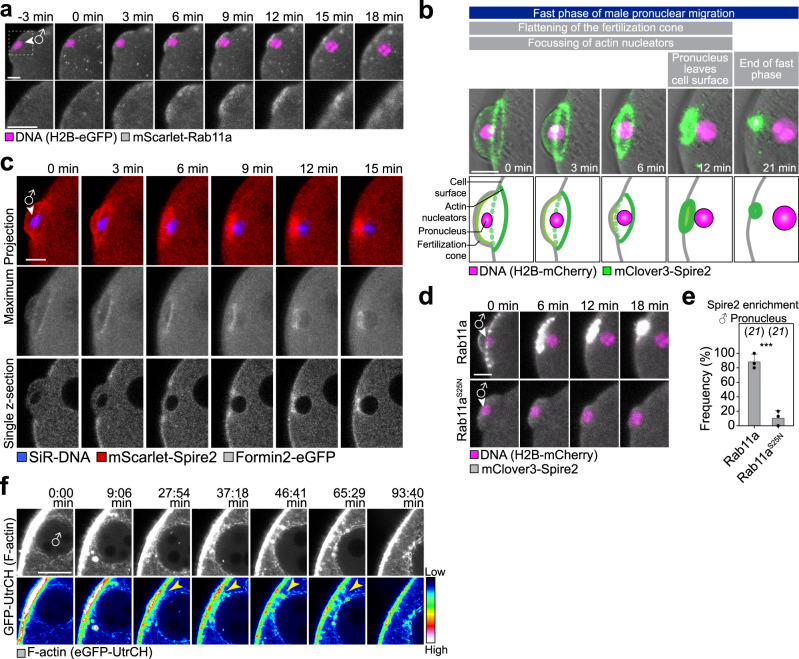
Rab11a, Spire, Formin-2 and actin accumulate in the fertilization cone. **a** Three-dimensional time-lapse images of mScarlet-Rab11a and male pronucleus (♂, H2B-EGFP) relative to pronuclear formation (0 min) in live zygotes (z-projection of seven sections, every 1.5 µm). The region outlined in the overview is magnified in the bottom panel. **b** Three-dimensional time-lapse images of mClover3-Spire2 and male pronucleus (♂) relative to pronuclear formation (0 min) in live zygotes (z-projection of 20 sections, every 1.5 µm). Below, schematic depicting the stages of the fast migration phase of pronuclei from time point of pronuclear formation until pronucleus is launched off the cell surface. **c** Three-dimensional time-lapse images of mClover3-Spire2, Formin-2-EGFP and male pronucleus (♂) relative to pronuclear formation (0 min) in live zygotes (top, z-projection of 20 sections, every 1.5 µm or bottom, single planes of middle slices of male pronucleus). **d** Three-dimensional time-lapse images of mClover3-Spire2 and male pronucleus (♂) in live zygotes expressing SNAP-Rab11a or SNAP-Rab11a^S25N^. **e** Histogram showing percentage of SNAP-Rab11a- or SNAP-Rab11^S25N^-expressing zygotes with cortical mClover3-Spire2 enrichment quantified from (**d**). Data are mean ± s.d. from three independent experiments. The total number of analyzed zygotes is specified in italics. Two-tailed Student’s *t* test was used to test for significance (*p* = 0.0007). **f** Super-resolution (airyscan) time-lapse images of F-actin (single section) displayed in grayscale (top) and fluorescence intensity map (bottom, pseudocolour, 12-Bit grayscale) in live zygotes in proximity of the forming male pronucleus (♂). Corresponding look-up table is shown as a colour scale bar ranging from intensity levels 1 (low) to 4096 (high). Time point 0 min marks the start of acquisition. Yellow arrowheads mark increased F-actin levels between cell cortex and male pronucleus. Scale bars, 10 μm.

Although dominant-negative Rab11a did not block pronuclear migration, it decreased the velocity of the male and female pronuclei during the initial fast migratory phase by ~60–70% (Fig. 3d, e). We thus investigated how Rab11a promotes this phase of migration. High-resolution live microscopy of mScarlet-Rab11a revealed an unexpected Rab11a-positive structure in proximity of the forming male pronucleus (Fig. 4a and Supplementary Fig. 4a): Rab11a was strongly enriched at the cell surface, directly behind the forming male pronucleus (Fig. 4a and Supplementary Movie 5). The enrichment was transient (lasting for 22 ± 15 min; *n* = 19), coinciding with the fast migration phase. In mouse oocytes, Rab11a-vesicles recruit the actin nucleation factor Spire2 to the plasma membrane^32^, which acts in concert with Formin-2 to generate actin filaments in mouse oocytes^35^. We thus asked if Spire2 and Formin-2 are also enriched behind the male pronucleus. Live-cell microscopy revealed that fluorescently tagged versions of the actin nucleation factors Spire2 and Formin-2 also became concentrated in the cell surface region behind the forming male pronucleus, concomitant with the enrichment of Rab11a (Fig. 4b, c, Supplementary Fig. 4b, c and Supplementary Movie 6).

High-resolution 3D microscopy revealed that Spire2 was enriched throughout the entire fertilization cone, and most highly concentrated in a ring at the base of the cone (Fig. 4b and Supplementary Fig. 4f). As the cone flattened, the Spire2 ring contracted and eventually coalesced into a single spot directly behind the male pronucleus.

The flattening fertilization cone moved the male pronucleus inwards. The pronucleus remained in direct proximity of the cell surface as the cone flattened. Only when the boundary of the contracting Spire2 ring reached the area just behind the male pronucleus, the male pronucleus detached and rapidly moved inwards (Fig. 4b and Supplementary Fig. 4f). Formin-2 was initially uniformly located at the cortex and rather depleted in the area of the fertilization cone (Fig. 4c and Supplementary Fig. 4g). As the fertilization cone flattened, Formin-2 became gradually enriched at the base of the contracting fertilization cone and was most highly concentrated behind the male pronucleus as it detached from the plasma membrane. The initial rapid phase of pronuclear migration is hence a two-step process: first, the flattening of the fertilization cone that moves the male pronucleus inwards and second, the launching of the male pronucleus away from the plasma membrane that coincides with an accumulation of the actin nucleation factors Spire2 and Formin-2 behind the male pronucleus.

Finally, we tested if the ablation of the initial fast phase of migration in dominant-negative Rab11a expressing oocytes may be due to a requirement of Rab11a in targeting the actin nucleation factors to the fertilization cone. Indeed, Spire2 was no longer enriched in the cell surface region in proximity of the forming male pronucleus upon expression of dominant-negative Rab11a (Fig. 4d, e).

### Actin nucleation by Spire2 and Formin-2 pushes the male pronucleus inwards

Consistent with an accumulation of actin nucleation factors, the cell surface region was an active site of actin nucleation, and F-actin-enriched patches were transiently detected below the cell surface as the pronucleus left the cell surface (Fig. 4f, Supplementary Movie 7 and Supplementary Fig. 4h). A previous study reported that the actin network density towards the cortex is higher than the density towards the centre in the region of the pronuclei^5^, consistent with this observation.

**Fig. 5:**
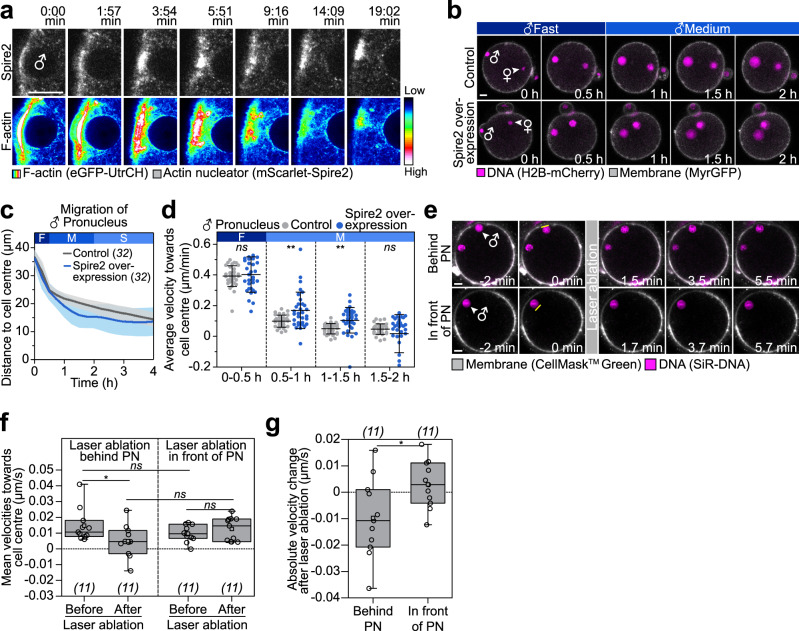
Spire-dependent local actin nucleation drives pronuclear migration in the periphery of the zygote. **a** Super-resolution (airyscan) time-lapse images of mScarlet-Spire2 (white) and F-actin (EGFP-UtrCH, pseudocolour, 12-Bit grayscale) in live zygotes in proximity of the forming male pronucleus displayed as single plane. Time point 0 marks the start of acquisition. Corresponding look-up table is shown as a colour scale ranging from intensity levels 1 (low) to 4096 (high). **b** Three-dimensional time-lapse images of pronuclei and the cell surface relative to pronuclear formation (0 h) in zygotes overexpressing SNAP (control) or SNAP-Spire2. Female (♀) and male (♂) genomes are shown. Phases with respective movement speeds are classified as in Fig. 1a. **c** The mean distance (thick line) of male pronuclei centroids to zygote centre during pronuclear migration in zygotes overexpressing SNAP or SNAP-Spire2 was calculated from (**b**). The total number of analyzed zygotes specified in italics was pooled from three independent experiments. S.d. shown as shaded areas. **d** Statistical plots of average velocities of male pronuclei during pronuclear migration in zygotes overexpressing SNAP (control) or SNAP-Spire2 calculated from (**c**) with the same sample size as in (**c**). Statistical plot shows mean ± s.d. Two-tailed Student’s *t* test was used to test for significance (from left to right: *p* = 0.7, *p* = 0.003, *p* = 0.001 and *p* = 0.2). **e** Laser ablation of live zygotes behind or in front of the male pronucleus (PN) during fast migration phase; yellow boxes indicate region of laser ablation. Single section time-lapse images of pronuclei (SiR-DNA) and the cell surface (CellMask Green) in live zygotes relative to time point of laser ablation (0 min) are presented. Images were acquired at 20-s intervals before and after laser ablation. **f** Box plot of velocities showing median (line), mean (small square), 5th, 95th (whiskers) and 25th and 75th percentile (box) with individual values (circles) calculated from Supplementary Fig. 5f as an average velocity of the 80 s time window before and after ablation from the total number of zygotes (italics) pooled from four independent experiments. Migration towards cell centre (>0) and towards cell surface (<0). Two-tailed Student’s *t* test was used to test for significance (from top to bottom: *p* = 0.2, *p* = 0.04, *p* = 0.06 and *p* = 0.3). **g** Box plot of velocity change after laser ablation showing median (line), mean (small square), 5th, 95th (whiskers) and 25th and 75th percentile (box) with individual values (circles) calculated from (**f**) with the same sample size as (**f**). Absolute velocities were calculated through subtraction of velocities before ablation from velocities after ablation. Increase (>0) or decrease (<0) of velocity after laser ablation. Two-tailed Student’s *t* test was used to test for significance (*p* = 0.03).

It is well established that local actin nucleation can generate pushing forces, which can for instance promote membrane protrusion or the propulsion of pathogens in a host cell^36–39^. We therefore asked if Spire and Formin-2 in the region of the fertilization cone might similarly cause pushing forces that propel the pronuclei away from the cell surface towards the centre of the zygote. To answer this question, we first overexpressed Spire2 to assess if we could enhance inwards pronuclear movement by increasing local actin nucleation. Indeed, overexpression of Spire2 increased F-actin behind the male pronucleus (Fig. 5a and Supplementary Movie 8), and strikingly, extended the rapid fast inwards movement of the male pronucleus, resulting in a 70–110% increase in the velocity of the male pronucleus between 0.5 and 1.5 h after its formation (Fig. 5b–d, Supplementary Fig. 5a and Supplementary Movie 9). In 14/31 zygotes that expressed very high levels of Spire2, the pronucleus moved beyond the zygote’s centre (Supplementary Movie 10), into the opposite hemisphere of the zygote. In five of these cells, the pronuclei even reached the opposite cortex and bounced back from there, coinciding with a new Spire2 enrichment event (Supplementary Fig. 5b and Supplementary Movie 11).

When Spire2 was highly overexpressed, some zygotes formed Spire2 surface patches in regions that were devoid of pronuclei. Interestingly, also these Spire2 surface patches generated inwards-directed forces, as we observed five cases, where oil droplets were located close to these patches and then rapidly accelerated inwards (Supplementary Fig. 5c and Supplementary Movie 12). Together, these data establish that local enrichments of Spire at the cortex cause inwards-directed forces that can move structures such as oil droplets and pronuclei away from the cell periphery. Consistent with inwards-directed forces in the region of the fertilization cone, particle image velocimetry (PIV) analysis revealed local inwards-directed flows in the region of the male pronucleus, but no global large-scale flows throughout the cytoplasm (Supplementary Fig. 5d).

To directly test for pushing forces, we carried out a laser ablation experiment: using a two-photon laser, we cut either behind or in front of the male pronucleus during the fast migration phase (Fig. 5e and Supplementary Fig. 5e). Cutting behind the pronucleus led to a significant ~70% decrease in pronuclear velocity (Fig. 5f, g). In contrast, cutting in front of the pronucleus, did not decrease its velocity (Fig. 5f, g). Together, these data establish that local pushing forces that act behind the male pronucleus move the pronucleus inwards during the initial fast migration phase.

**Fig. 6:**
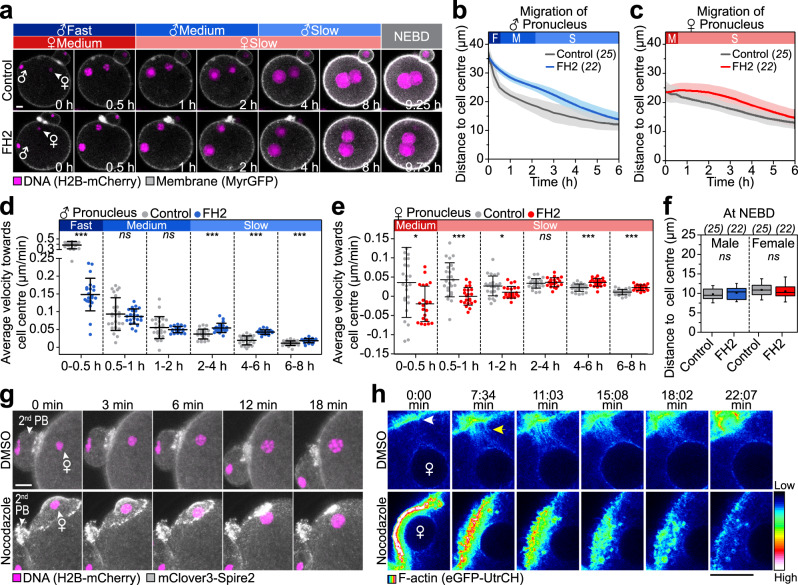
The fast migration mode requires actin nucleation in the periphery of the zygote. **a** Three-dimensional time-lapse images of pronuclei and the cell surface relative to pronuclear formation (0 h) in zygotes injected with 3 μM MBP (control) or FH2 until nuclear envelope breakdown (NEBD). Female (♀) and male (♂) genomes are shown. Phases with respective movement speeds are classified as in Fig. 1a. The mean distance (thick line) of male (**b**) and female (**c**) pronuclei centroids to zygote centre during pronuclear migration in zygotes injected with MBP or FH2 was calculated from (**a**). The total number of analyzed zygotes specified in italics was pooled from three independent experiments. S.d. shown as shaded areas. Statistical plots of average velocities of male (**d**) and female (**e**) pronuclei during pronuclear migration in zygotes injected with MBP or FH2 calculated from (**b**) and (**c**) with the same sample sizes as in (**b**) and (**c**), respectively. Statistical plots show mean ± s.d. Two-tailed Student’s *t* test was used to test for significance (from left to right: **d** *p* < 0.0001, *p* = 0.5, *p* = 0.4, *p* < 0.0001, *p* < 0.0001 and *p* = 0.0003; **e** *p* = 0.01, *p* < 0.0001, *p* = 0.01, *p* = 0.5, *p* < 0.0001 and *p* < 0.0001). **f** Distance of pronuclei centroids to zygote centre at NEBD for zygotes injected with MBP or FH2 calculated from data sets in (**a**). Box plot showing median (line), mean (small square), 5th, 95th (whiskers) and 25th and 75th percentile (box). Two-tailed Student’s *t* test was used to test for significance (male *p* = 0.4 and female *p* = 0.5). **g** Three-dimensional time-lapse images of with cortical mClover-Spire2 and female pronuclei in DMSO- or nocodazole-treated zygotes relative to pronuclear formation (0 min) in live zygotes (z-projection of 20 sections, every 1.5 µm). Second polar bodies (PB) are labelled. **h** Super-resolution (airyscan) time-lapse images of mScarlet-Spire2 (white) and F-actin (EGFP-UtrCH, pseudocolour, 12-Bit grayscale) in live zygotes treated with DMSO or nocodazole in proximity of the forming female pronucleus displayed as single plane. Time point 0 marks the start of acquisition. Corresponding look-up table is shown as a colour scale bar ranging from intensity levels 1 (low) to 4096 (high). The brighter beans radiating from the cytokinetic furrow (white arrow) of the second polar body extrusion in DMSO-treated zygotes are actin filaments located in the spindle remnant (yellow arrow) and appear distinct from the places that emerge below the cell surface in nocodazole-treated zygotes. Scale bars, 10 µm.

Next, we tested directly if actin nucleation by Spire and Formin-2 is required for the initial rapid phase of pronuclear migration in proximity of the cell surface. To this end, we used a dominant-negative Formin Homology 2 domain (FH2) construct, which blocks the interaction between Spire and Formin-2^40^. Previously, we showed that this FH2 construct inhibits Spire- and Formin-2-dependent nucleation of the cytoplasmic actin network in oocytes^40^, and we here show that it also depletes the cytoplasmic actin network in zygotes (Supplementary Fig. 6c, e). Microinjection of recombinant FH2 domain significantly reduced the mean velocity by 61% during the initial 30 min of male pronuclear migration (Fig. 6a, b, d). Pronuclear migration was not inhibited during the slower, second phase (Fig. 6d), and the pronuclei eventually reached the cell centre (Fig. 6f).

As we had observed that the female pronucleus also adopts a more pronounced rapid initial migratory mode when it is forced to form close to the cell surface (Fig. 2a–d), we next asked if this rapid mode is also due to a local enrichment of Rab11a and Spire2. Indeed, Rab11a and Spire2 were both enriched behind the female pronucleus when it formed beneath the cell surface, in a structure that closely resembled the fertilization cone (Fig. 6g and Supplementary Fig. 4d, e, i). As the fertilization cone-like structure flattened, the female pronucleus moved inwards and prominent actin patches appeared between the cell cortex and the female pronucleus (Fig. 6h and Supplementary Movie 13). The launching of the peripheral female pronucleus was hence very similar to that of the male pronucleus.

Also in control cells, the female pronucleus was slowed down by FH2 (Fig. 6c, e) or dominant-negative Rab11a during the initial phase of migration (Fig. 3c, e). Rab11a and Spire/Formin-2-dependent actin nucleation hence also promotes the early stages of female pronuclear migration in unperturbed cells. Actin and Spire2 were strongly enriched in the cytokinetic furrow (Fig. 6g, h), and also the spindle remnant contained actin (Fig. 6h, top row)^41^. Both structures are in close proximity of the female pronucleus. An additional prominent enrichment of Rab11a and Spire/Formin-2 at the cortex was not detectable but might have been shielded by their strong enrichment in the cytokinetic furrow.

Together, these data establish a two-step mechanism that drives the rapid inwards pronuclear movement in the cortical region of the zygote. The male pronucleus forms within the fertilization cone, which becomes enriched in Spire and Formin-2 in a Rab11a-dependent manner. First, the cone flattens, thereby moving the pronucleus inwards. Second, Spire and Formin-2 concentrate behind the male pronucleus where they locally nucleate actin. This generates inwards-directed forces that launch the pronucleus away from the cell surface. The female pronucleus can trigger the same launching mechanism when it is forced to form directly underneath the cell surface and is also accelerated inwards by Spire and Formin-2 in unperturbed zygotes, albeit to a lesser degree than the male pronucleus.

### Microtubules and dynein drive the slower, second phase of pronuclear migration

**Fig. 7:**
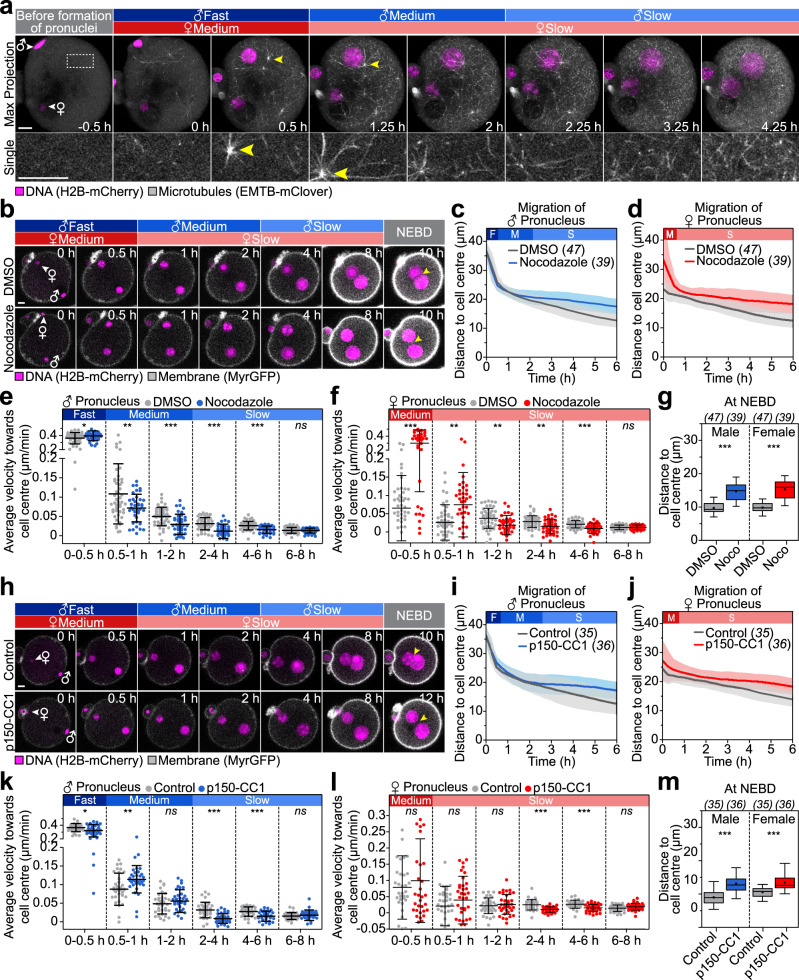
Microtubule- and dynein-dependent transport drives pronuclear migration throughout the centre. **a** 3D super-resolution (airyscan) time-lapse images of microtubules (EMTB-mClover) and pronuclei relative to pronuclear formation (0 h) in live zygotes (z-projection of 5 (EMTB) or 11 (H2B) sections, every 4 µm). Female (♀) and male (♂) genomes are labelled. Phases of respective movement speeds are classified as in Fig. 1a. Magnified region outlined in the overview presented as single section. Cell centre directed aster movement (yellow arrowheads). **b** 3D time-lapse images of pronuclei and the cell surface relative to pronuclear formation (0 h) in zygotes treated with DMSO or 10 µM nocodazole. Yellow arrowheads highlight gap between pronuclei at nuclear envelope breakdown (NEBD). The mean distance (thick line) of male (**c**) and female (**d**) pronuclei centroids to zygote centre in DMSO- or nocodazole-treated zygotes was calculated from (**b**). The total number of analyzed zygotes (italics) was pooled from four independent experiments. S.d. shown as shaded areas. Statistical plots of average velocities of male (**e**) and female (**f**) pronuclei in DMSO- or nocodazole-treated zygotes calculated from (**c**) and (**d**) with the same sample sizes as in (**c**) and (**d**), respectively. Statistical plots show mean ± s.d. Two-tailed Student’s *t* test was used to test for significance (from left to right: **e** *p* = 0.04, *p* = 0.005, *p* = 0.0004, *p* < 0.0001, *p* < 0.0001 and *p* = 0.7; **f** *p* < 0.0001, *p* = 0.003, *p* = 0.002, *p* = 0.001, *p* < 0.0001 and *p* = 0.8). **g** Distance of pronuclei centroids to zygote centre at NEBD in DMSO- or nocodazole-treated zygotes. Box plot showing median (line), mean (small square), 5th, 95th (whiskers) and 25th and 75th percentile (box). Two-tailed Student’s *t* test was used to test for significance (male *p* < 0.0001 and female *p* < 0.0001). **h** Three-dimensional time-lapse images of pronuclei and the cell surface in zygotes microinjected with 30 µM MBP (control) or p150-CC1. Labels as (**b**). The mean distance of male (**i**) and female (**j**) pronuclei centroids to zygote centre in zygotes microinjected with MBP or p150-CC1 was calculated from (**h**) from total number of analyzed zygotes (italics) pooled from four independent experiments. S.d. shown as shaded areas. Statistical plots of average velocities of male (**k**) and female (**l**) pronuclei during pronuclear migration in zygotes microinjected with MBP or p150-CC1 calculated from (**i**) and (**j**) with the same sample size as in (**i**) and (**j**), respectively. Statistical plots show mean ± s.d. Two-tailed Student’s *t* test was used to test for significance (from left to right: **k** *p* = 0.02, *p* = 0.007, *p* = 0.3, *p* < 0.0001, *p* = 0.0002 and *p* = 0.4; **l** *p* = 0.4, *p* = 0.3, *p* = 0.5, *p* < 0.0001, *p* = 0.0001 and *p* = 0.06). **m** Distance of pronuclei centroids to zygote centre at NEBD in zygotes microinjected with MBP (control) or p150-CC1 calculated from (**h**). Box plot showing median (line), mean (small square), 5th, 95th (whiskers) and 25th and 75th percentile (box). Two-tailed Student’s *t* test was used to test for significance (male *p* < 0.0001 and female *p* < 0.0001). Scale bars, 10 μm.

We next investigated the slow movement pattern in the central region of the zygote. Neither loss of vesicles by dominant-negative Rab11a nor disruption of the cytoplasmic actin network by FH2 slowed down pronuclei in the central region of the zygote (Figs. 3a–f and 6a–f). Moreover, Rab11a-positive vesicles were strongly depleted from oocytes during the second phase of migration (Supplementary Fig. 2a, b). Together, these data suggest that the second migration phase does not rely on Rab11a-positive vesicles or the cytoplasmic actin network but must be mediated by other cytoskeletal structures instead. Consistent with previous studies^23,42^, zygotes assembled a cytoplasmic microtubule network as the pronuclei formed and grew (Fig. 7a and Supplementary Movie 14). Live microscopy of microtubules in zygotes using the fluorescently tagged microtubule binding domain of MAP7/Ensconsin (EMTB) revealed that the microtubule network density increased gradually from formation of the pronuclei onwards (Fig. 7a and Supplementary Fig. 7a). The microtubule network was dynamic and included multiple microtubule asters that were nucleated at aMTOCs (Fig. 7a)^42^. We hence asked if microtubules are involved in the second, slower phase of migration that occurs throughout the centre of the zygote.

Depolymerization of microtubules by nocodazole (Supplementary Fig. 7b) significantly decreased the speed of both the male and the female pronucleus during the second slower phase of migration (Fig. 7b–f and Supplementary Movie 15). Consistently, pronuclear migration was incomplete, because the distance of the male and female pronucleus from the cell centre at NEBD was increased by 50% and 60%, respectively (Fig. 7g). This demonstrates that both pronuclei rely on microtubules to efficiently migrate to the zygote’s centre. By contrast, the fast migration in the periphery of the zygote was not slowed down (Fig. 7c, e), indicating that microtubules are dispensable for the initial fast migration phase.

Next, we investigated, which motor proteins are required for the inwards movement of the pronuclei. Pronuclear migration in several other species is dynein dependent^13,15,43^, making dynein a good candidate for inwards movement. Indeed, inhibition of dynein by microinjection of the p150-CC1 peptide^44^ significantly decreased the velocity of the male and female pronucleus during the second period of migration (Fig. 7h–l), while significantly increasing the distance of the pronuclei to the cell centre at NEBD was significantly increased (Fig. 7m).

In further support of inwards-directed forces acting within the microtubule network, 33/47 aMTOC-nucleated microtubule asters (nine cells) moved inwards over 10 h in a microtubule-dependent manner (Fig. 7a, Supplementary Fig. 7e–g and Supplementary Movie 14). Dynein inhibition also blocked the inwards-directed aMTOC movement, and even caused aMTOCs to drift outwards (Supplementary Fig. 7h, i). Moreover, the microtubule network was much less dynamic in 4/4 zygotes microinjected with the p150-CC1 peptide (Supplementary Fig. 7j and Supplementary Movie 16). However, the inwards-directed movement of the male and female pronucleus could occur independently of aMTOCs though, as their removal by Trim-away of Pericentrin^45,46^ (Supplementary Fig. 8a–d) did not affect pronuclear migration (Supplementary Fig. 8e–h) or the formation of the microtubule network (Supplementary Fig. 8c, i). Together, these data establish that the second, slow phase of migration is driven by microtubules and dynein.

### Microtubule network dynamics during the second phase rely on an intact actin cytoskeleton

This finding might be surprising given that current models for pronuclear migration are focused on actin instead of microtubules^5,25,26^. The importance of actin in pronuclear migration has been highlighted by work in which zygotes were treated with the actin depolymerizing drug cytochalasin D^5,25,27^. Indeed, cytochalasin D completely blocks pronuclear migration, as confirmed by our experiments (Supplementary Fig. 9a–f). As we outlined above, local actin polymerization in the region of the fertilization cone is essential for the initial fast mode of pronuclear migration, and it is clear why depolymerizing actin disrupts this initial mode. But why is the second, slow phase also disrupted by cytochalasin D, if it is microtubule dependent?

**Fig. 8:**
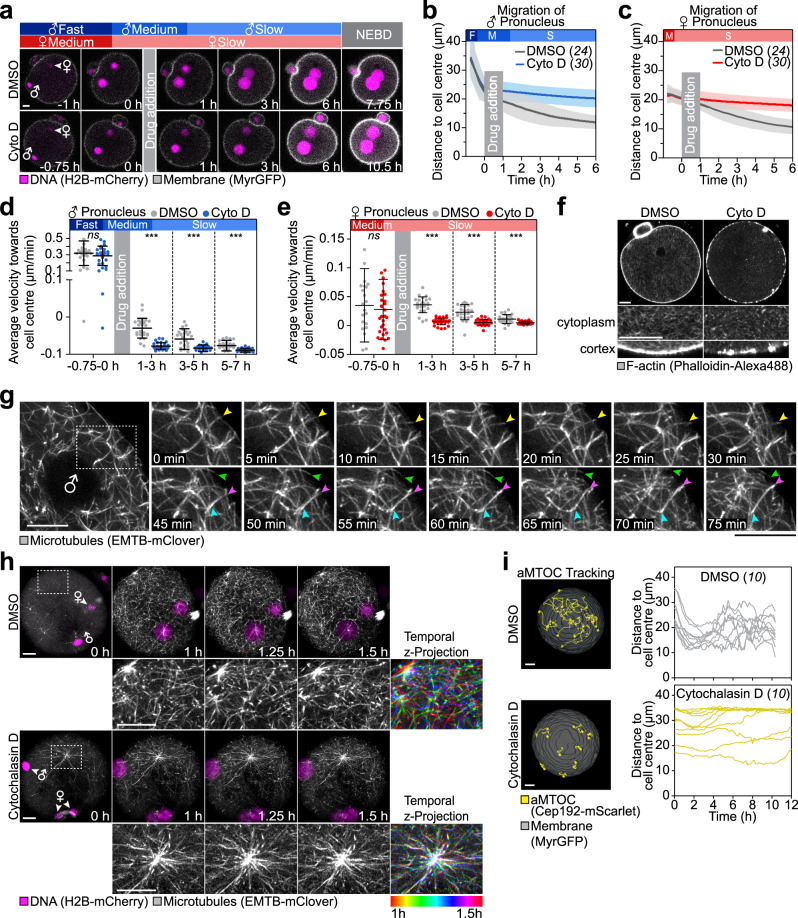
Inwards-directed microtubule-based transport requires actin. **a** 3D time-lapse images of pronuclei and the cell surface in zygotes acutely treated with DMSO or 5 µg/ml cytochalasin D in slow migration phase relative to time point of drug addition (0 h). Female (♀) and male (♂) genomes are shown at pronuclear formation until nuclear envelope breakdown (NEBD). Phases of respective movement speeds are classified as in Fig. 1a. The mean distance (thick line) of male (**b**) and female (**c**) pronuclei centroids to zygote centre during pronuclear migration in zygotes acutely treated with DMSO or cytochalasin D from (**a**). Total number of analyzed zygotes (italics) was pooled from three independent experiments. S.d. shown as shaded areas. Statistical plots of average velocities of male (**d**) and female (**e**) pronuclei during pronuclear migration in zygotes acutely treated with DMSO or cytochalasin D calculated from (**b**) and (**c**) with the same sample size as in (**b**) and (**c**), respectively. Statistical plots show mean ± s.d. Two-tailed Student’s *t* test was used to test for significance (from left to right: **d** *p* = 0.4, *p* < 0.0001, *p* = 0.0003 and *p* = 0.0004; **e** *p* = 0.7, *p* < 0.0001, *p* < 0.0001 and *p* = 0.0009). **f** Super-resolution (airyscan) images of zygotes treated with DMSO or cytochalasin D, fixed 5 hpi and stained for F-actin with Phalloidin-Alexa488. Magnified regions of the cytoplasmic actin network and the actin cortex are shown. **g** Super-resolution (airyscan) time-lapse images of microtubules (EMTB-mClover3, z-projection of three sections, every 1.5 µm) in live zygotes. The magnified region presented as time-lapse starting ~5.5 hpi (0 min). Arrowheads highlight microtubules encountering the cell cortex resulting in displacement away from cortex (pink, blue) or buckling (yellow, green). **h** 3D super-resolution (airyscan) time-lapse images of microtubules and pronuclei relative to pronuclear formation (0 h) in live zygotes treated with DMSO or cytochalasin D (z-projection as Fig. 7a). Three time points from 1 to 1.5 h (15-min intervals) were superimposed using ImageJ’s Temporal-Colour Coder and represented in pseudocolour with the corresponding look-up table shown as a colour scale bar from time points 1 to 1.5 h. **i** Right, 3D tracking of aMTOCs (Cep192-mScarlet) in zygotes relative to pronuclear formation (0 h) in zygotes treated with DMSO or cytochalasin D. Left, graphs display tracks of acentriolar microtubule-organizing centres (aMTOCs) in respect to cell centre. The total number of analyzed aMTOCs is specified in italics each from one representative cell per condition. Scale bars, 10 μm.

We first tested if cytochalasin D might prevent an early essential step in pronuclear assembly or migration but could in fact be dispensable for pronuclear migration in the second phase. To this end, we added cytochalasin D acutely, after the pronuclei had formed and started to migrate. Cytochalasin D still prevented pronuclear migration though (Fig. 8a–e), suggesting that actin does indeed play an essential role during the second slow phase of migration as well.

Which actin structures are required for the second, slow phase of migration? Our data suggested that the cytoplasmic actin network, which forms on Rab11a-vesicles and is Spire- and Formin-2-dependent, is not essential during this phase (Figs. 3a–e and 6a–e). Zygotes also contain another prominent actin structure that was disrupted by cytochalasin D but not by the FH2 construct—the actin cortex (Fig. 8f and Supplementary Figs. 9g and 6c, d). There is indeed evidence that the actin cortex promotes pronuclear migration in other systems. In worm zygotes, pronuclear migration and spindle centration are thought to rely on pushing of microtubules against the cell cortex^20,47,48^. Disrupting actin reduces the efficiency of pushing^20,49–52^.

Could pushing against the actin cortex also be important in mouse zygotes? High-resolution imaging of microtubules in live mouse zygotes revealed that microtubules continued to grow as they encountered the cell surface (Fig. 8g and Supplementary Movie 17). Upon hitting the surface, they bent (yellow and green arrowhead, Fig. 8g), or the microtubule end pointing towards the centre of the zygote was pushed inwards^53^ (magenta and blue arrowheads, Fig. 8g). We also found that microtubule asters nucleated at aMTOCs had microtubules that reached all the way to the cortex and grew against the cortex as the aster moved inwards, consistent with a pushing-based mechanism (Supplementary Fig. 9h and Supplementary Movie 18).

If microtubules, pronuclei and aMTOCs rely on pushing against the cortex to move inwards, disrupting cortical actin should interfere with their movement. To test this possibility, we imaged microtubules and aMTOCs in the presence and absence of cytochalasin D. Strikingly, the microtubule network was static in 7/7 cytochalasin D-treated zygotes (Fig. 8h and Supplementary Movie 19). Moreover, microtubule asters and aMTOCs failed to migrate inwards upon actin depolymerization (Fig. 8h, i and Supplementary Fig. 9j), just like pronuclei (Fig. 8a–c and Supplementary Fig. 9a–c). Taken together, our data show that an intact actin cytoskeleton is essential for physiological microtubule network dynamics as required for the second microtubule-dependent phase of pronuclear migration.

**Fig. 9:**
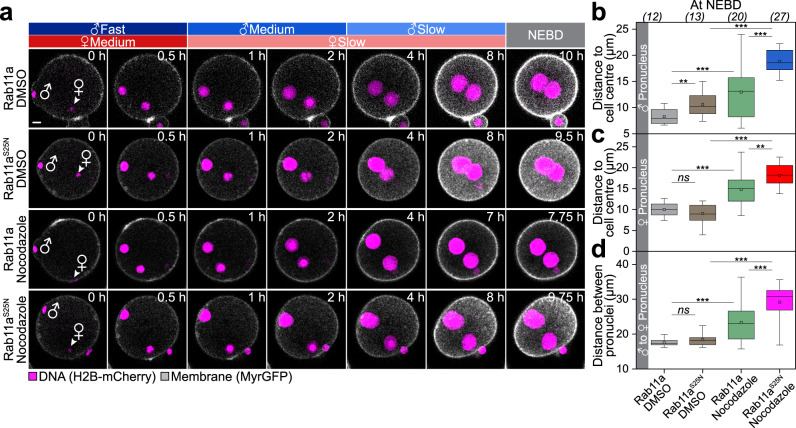
Local actin nucleation by Spire and Formin-2 and microtubule-based transport drive pronuclear migration in a partially redundant manner. **a** Three-dimensional time-lapse images of pronuclei and the cell surface relative to pronuclear formation (0 h) in zygotes expressing SNAP-Rab11a or SNAP-Rab11a^S25N^ and treated with DMSO or 10 µM nocodazole, respectively. Female (♀) and male (♂) genomes are shown at pronuclear formation until nuclear envelope breakdown (NEBD). Phases of respective movement speeds are classified as in Fig. 1a. In 10/27 (37%) of Rab11a^S25N^ nocodazole zygotes, we observed that cells adopted a severely squeezed shape during pronuclear migration pushing pronuclei located beneath the cell surface closer to cell centre, which may account for some movement of pronuclei indirectly to cell centre. Distance of male (**b**) and female (**c**) pronuclei centroids to zygote centre at NEBD in zygotes expressing SNAP-Rab11a or SNAP-Rab11a^S25N^ and treated with DMSO or 10 µM nocodazole, respectively, calculated from (**a**). Total number of analyzed zygotes (italics) was pooled from three independent experiments. Box plot showing median (line), mean (small square), 5th, 95th (whiskers) and 25th and 75th percentile (box). Two-tailed Student’s *t* test was used to test for significance (from top to bottom: **b** *p* < 0.0001, *p* < 0.0001, *p* = 0.0006 and *p* = 0.005; **c** *p* < 0.0001, *p* = 0.001, *p* < 0.0001 and *p* = 0.2). **d** Distance between male and female pronuclei centroids at NEBD in zygotes expressing SNAP-Rab11a or SNAP-Rab11a^S25N^ and treated with DMSO or 10 µM nocodazole, respectively, calculated from (**a**). Box plot showing median (line), mean (small square), 5th, 95th (whiskers) and 25th and 75th percentile (box). Two-tailed Student’s *t* test was used to test for significance (from top to bottom: *p* < 0.0001, *p* = 0.0008, *p* = 0.0002 and *p* = 0.2). Scale bars, 10 μm.

Our work establishes that two partially redundant mechanisms drive pronuclear migration in the mouse zygote: a mechanism that involves the flattening of the fertilization cone and relies on local actin polymerization by Spire and Formin-2 that acts in the periphery of the zygote, and a microtubule-dependent mechanism that acts in the central region of the zygote. Inhibition of either mechanism alone does not entirely prevent pronuclear migration or the unification of the parental genomes (Figs. 3a–f, 6a–f, 7b–g and Supplementary Table 1, column 8), suggesting that both mechanisms act in a redundant manner to promote pronuclear migration. In further support of redundance, the simultaneous inhibition of the microtubule-dependent pathway (by nocodazole) and the Rab11a-Spire-Fmn2-dependent pathway (by Rab11a^S25N^) (Fig. 9a, Supplementary Fig. 10a, b and Supplementary Movie 20) prevented pronuclear migration in 11/27 zygotes (Fig. 9a, bottom panel) and led to an additional significant increase in the distance of the pronuclei from the zygote’s centre at NEBD (by 120 or 80% in comparison to the controls, in male and female pronuclei, respectively (Fig. 9b, c)), despite compression of 10/27 zygotes which caused a decrease in the apparent distance of the pronuclei from the cell centre (Fig. 9a, bottom panel). Simultaneous inhibition of both pathways with cytochalasin D also caused the pronuclei to stay at a large distance from each other at NEBD (Fig. 9d and Supplementary Fig. 9f) and lead to the formation of two entirely separate spindles (Supplementary Table 1, column 8). Together, these data establish that the two pathways that drive pronuclear migration in mouse zygotes are partially redundant.

## Discussion

**Fig. 10:**
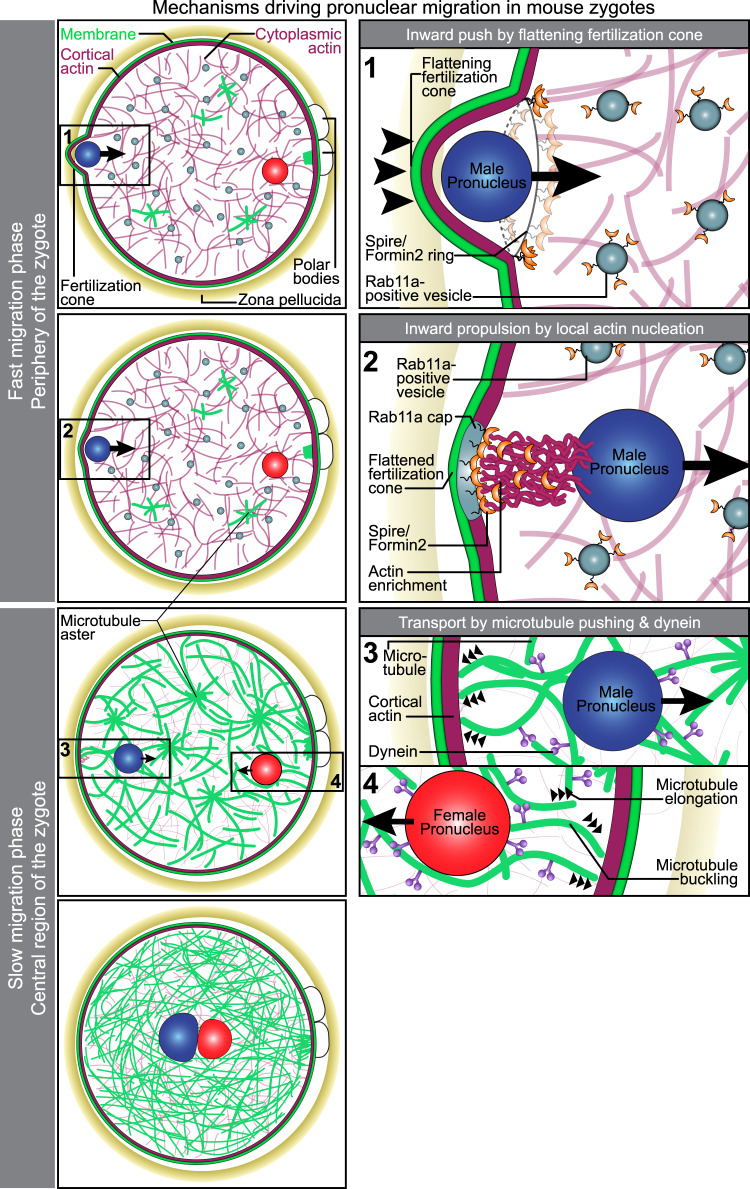
Model for pronuclear migration in mouse zygotes. Pronuclear migration is initiated by the flattening of the cone, which moves the male pronucleus inwards. During this stage, actin nucleation factors Formin-2/Spire concentrate as a ring structure at the edge of the shrinking fertilization cone. Next, actin nucleation by the Formin-2/Spire, which are targeted to the fertilization cone by Rab11a-positive vesicles, launches the pronuclei away from the cell surface. Female pronuclei typically assemble further away from the cell surface due to the metaphase II spindle remnant (green microtubule clusters) and their fast migration phase is less pronounced. In the slow migration phase (small arrowheads) microtubules and dynein drive the inwards-directed movement of the two pronuclei. Both mechanisms cooperate in a partially redundant manner to centre the two pronuclei.

In this study, we established methods to follow and experimentally probe the entire process of pronuclear migration in mammalian zygotes. Using these methods, we demonstrate that pronuclear migration is driven by two partially redundant mechanisms and define the molecular basis for these two mechanisms (Fig. 10).

The first mechanism is required to launch the pronuclei away from the cell cortex. This mechanism is most important for the male pronucleus because it forms within the fertilization cone, from where it is rapidly accelerated inwards by the flattening fertilization cone. The inwards-directed movement is enhanced by pushing from behind the male pronucleus and depends on local actin nucleation by Spire and Formin-2, which become enriched in the fertilization cone in a Rab11a-dependent manner. This mechanism is highly effective in launching the male pronucleus away from the zygote’s surface, as illustrated by the fact that enhancing actin nucleation from the surface can push pronuclei even beyond the zygote’s centre, into the opposite hemisphere of the zygote (Supplementary Movie 10). The same mechanism accelerates the female pronucleus inwards, but to a lesser degree as the female pronucleus normally forms further away from the cell surface.

The second mechanism is required to actually centre the pronuclei. In this mechanism, microtubules and dynein move the pronuclei inwards until they meet in the zygote’s centre. This mechanism likely relies on an intact actin cortex, explaining why depolymerization of actin with drugs like cytochalasin D fully blocks pronuclear migration^5,25–27^.

Why are zygotes employing flattening of the fertilization cone and local actin nucleation by Spire and Formin-2 in addition to the more classical microtubule-dependent mechanism to drive pronuclear migration? There are several possible answers to this question.

First, it may make the mechanism of pronuclear migration more robust and increase the chance that both pronuclei are in immediate proximity of each other when they disassemble at mitotic entry. Our data establish that actin and microtubules act in concert to unite the paternal and maternal chromosomes in mouse zygotes. When microtubules are depolymerized or dynein is inhibited, pronuclei still move inwards, albeit at a slower speed, and are positioned significantly further apart at NEBD (Fig. 7g, m). This suggests that the Rab11a-Spire-Formin-2 mechanism can at least partially rescue pronuclear migration in the absence of microtubules. Conversely, microtubules are able to move pronuclei towards the zygote centre when the Rab11a-Spire-Formin-2 pathway is disrupted. The unification of the parental genomes hence appears to be doubly protected, by the Rab11a-Spire-Formin-2-dependent pathway that acts predominantly in the periphery, and the dynein- and microtubule-dependent mechanism throughout the central region of the zygote.

Interestingly, our data suggest that pronuclei always break down at roughly the same time after their formation (Supplementary Figs. 3k and 7k), independently of whether they successfully migrated inwards (Figs. 3f and 7g). There hence appears to be no checkpoint that monitors pronuclear position, which might be one of the reasons why this process is doubly protected.

Second, the flattening of the fertilization cone and the Rab11a-Spire-Formin-2 mechanism may help to initiate the centration of the pronuclei, while the microtubule system is reorganizing from a meiotic-like state, characterized by the presence of the spindle remnant at the end of meiosis II, to an interphase-like state, characterized by the presence of a microtubule network. The Rab11a-Spire-Formin-2 mechanism could already start to centre the pronuclei, while microtubules are still being reorganized, and thereby accelerate the unification of the parental genomes.

Third, moving the pronuclei away from the cortex before the interphase network is assembled may even be a prerequisite for efficient microtubule pushing and centration of pronuclei. In order for microtubules to push the pronuclei inwards they need to form between the pronucleus and the cell cortex. The initial actin-dependent propulsion of pronuclei may therefore help to generate the space that is required for efficient microtubule pushing.

Finally, it may help to synchronize the movement of the male and female pronucleus. Our data establish that the female pronucleus forms further inwards than the male pronucleus. Moving the male pronucleus rapidly inwards allows for it to catch up, and to reach the zygote’s centre as efficiently as the female pronucleus, as illustrated by Fig. 1b. In support of this notion, we observed that at NEBD the distance of male pronuclei to the cell centre was more severely increased (by 30%) than that of the female pronucleus (by 10%), when the fast actin-dependent launching mechanism of male pronuclei was disabled by expression of dominant-negative Rab11a^S25N^ (Fig. 3f).

It will be interesting to investigate if pronuclei are also rapidly pushed inwards by flattening of the fertilization cone and local actin-dependent pushing from the surface in other systems.

Generally, microtubules are thought to be the drivers of pronuclear centration. Yet, blocking actin nucleation delays male pronuclear migration in worm zygotes^20^. An initial, rapid actin-dependent migratory phase that accelerates pronuclei away from the surface may hence be conserved beyond mouse zygotes.

Defects during pronuclear migration lead to the failure of spindle alignment and impair embryonic development^4^. Our study defines two mechanisms that act in concert to achieve this vital task: a Rab11a-, Spire- and Formin-2-dependent mechanism that involves flattening of the fertilization cone and pushes the male pronucleus away from the cell surface by local actin nucleation, and a microtubule–dynein-dependent mechanism that acts throughout the centre to move both pronuclei inwards.

## Methods

### Preparation, culture and in vitro fertilization of mouse eggs

The maintenance and handling of all mice were performed in the MPI-BPC animal facility according to international animal welfare rules (Federation for Laboratory Animal Science Associations guidelines and recommendations). Requirements of formal control of the German national authorities and funding organizations were satisfied, and the study received approval by the Niedersächsisches Landesamt für Verbraucherschutz und Lebensmittelsicherheit (LAVES). 8–12-week-old C57BL/6J × CBA/CaOlaHsd F1 females (housing conditions: 21 °C ambient temperature, 52–55% humidity and a 14-h light/10-h dark cycle) were superovulated by injection of 5 or 7.5 IU pregnant mare serum gonadotropin (THP Medical Products, #hor-272-a) followed 48 h later by injection of 5 or 7.5 IU human chorionic gonadotropin (Intervet, Ovogest^®^ 1000). Thirteen hours later, meiosis II arrested eggs were collected from oviducts, cultured and microinjected in HEPES-buffered MEM-α medium (Gibco, #12000-014) supplemented with 5% foetal bovine serum (FBS, Gibco, #16000-044), 4 mM NaHCO_3_ (Sigma), 20 mM HEPES (Gibco) and 0.075 g/l potassium penicillin-G (Sigma) and 0.05 g/l streptomycin sulfate (Sigma). Microinjections of eggs were performed as previously described for prophase-arrested mouse oocytes^29,54^, but using thinner needles to penetrate the egg membrane more easily without using a piezo^55^ yet with high survival rates (83 ± 7%). Briefly, eggs were microinjected in homemade microinjection chambers using mercury-filled needles for quantitative microinjections. The homemade microinjection chambers were assembled by sticking a small rectangular piece of a coverslip on top of a standard size coverslip with a double-sided sticky tape, as previously described^29^. In this way, a 100 µm height injection shelf was generated, into which the oocytes were loaded during microinjection. The injected volumes ranged between 5 and 9 pl (3–5% of the egg volume). Concentrations of microinjected mRNAs were calculated by dividing the total volume of injected mRNA by the total volume of the egg (≈200 pl) and are listed here: 5–10 µg/ml MyrGFP, 0.2–0.5 µg/ml H2B-mCherry, 20 µg/ml EGFP-LaminB1, 10 µg/ml Rab11a constructs (SNAP-Rab11a, SNAP-Rab11a^S25N^ and mScarlet-Rab11a), 5 µg/ml Spire2 constructs for mild overexpression (mClover3-Spire2, SNAP-Spire2, mScarlet-Spire2) or 25 µg/ml EGFP-Spire2 for high overexpression, 90 µg/ml Formin-2-GFP, 80 µg/ml GFP-UtrCH, 60 µg/ml EMTB-mClover, 20 µg/ml Cep192-mScarlet and 6 µg/ml miRFP670-Trim21.

For TRIM-away of Pericentrin, the antibodies mouse IgG (Sigma, #12-371) and anti-pericentrin (BD Biosciences, #611815) were concentrated in PBS at 14,000 × *g* using Amicon^®^ Ultra centrifugal filters (Millipore, #UFC51024)^46^. Prior to microinjection, antibodies were diluted to 1 mg/ml in PBS containing a final concentration of 0.03% NP40. Antibodies were microinjected into eggs to a final concentration of 20 µg/ml simultaneously with mRNAs.

After microinjection, eggs were washed into CO_2_-dependent MEM-α medium supplemented with 5% FBS, 26 mM NaHCO_3_ and 0.075 g/l potassium penicillin-G (Sigma) and 0.05 g/l streptomycin sulfate (Sigma) and cultured at 37 °C in 5% CO_2_ for 3–4 h to allow recombinant protein expression. Sperm was collected from >12-week-old C57BL/6J × CBA/CaOlaHsd males and capacitated for 2 h in HTF medium (Millipore, #MR-070-D) supplemented with 2 mM Hypotaurine (Sigma, H1384). Subsequently, IVF of eggs was performed in a 40 µl droplet of HTF medium by adding 2–5 µl of capacitated sperm. After 2 h of IVF, zygotes were washed into HEPES-buffered M2 medium and immediately prepared for live-cell microscopy (start of acquisition within 1–1.5 h post IVF to capture pronuclear formation (Supplementary Fig. 1l) or immunofluorescence).

We confirmed that microinjection into eggs prior to IVF did not prevent efficient fertilization (Supplementary Fig. 11a, c) and supported healthy early development (Supplementary Fig. 11b, d, e).

### Preparation of zygotes for live microscopy or immunofluorescence

For drug additions, zygotes were washed into M2 medium containing drugs immediately after IVF. Nocodazole was added just prior to pronuclear formation (about 1 h after IVF) to prevent dispersion of maternal chromosomes. Zygotes were treated with the following drugs: 5 µg/ml cytochalasin D (Sigma, #C8273, dissolved in DMSO at 5 mg/ml), 1 or 10 µM nocodazole (Sigma, #M1404, dissolved in DMSO at 10 mM freshly prepared) and 4 µg/ml BFA (Sigma, #B6542, dissolved in Methanol at 1 mg/ml). For acute cytochalasin D treatment, zygotes were first imaged in M2 medium for 2–2.5 h before washed into M2 medium containing 5 µg/ml cytochalasin D.

For dynein inhibition experiments, zygotes were microinjected with 33 mg/ml purified p150-CC1-6xHis (41 kDa) or MBP-6xHis (43 kDa) in PBS containing 0.03% NP40 and 5 µg/ml Dextran Alexa Flour 680 (Invitrogen, D34680) to a final concentration of 30 µM protein per zygote. Alexa Fluor 680-labelled Dextran was used to verify that protein has entered zygotes. Confirming activity of p150-CC1, meiotic spindles in eggs become abnormally elongated and onset of NEBD was significantly delayed.

For Formin-2 inhibition, zygotes were microinjected with 5–6 mg/ml purified FH2-6xHis (51 kDa) or MBP-6xHis (43 kDa) in buffer (20 mM potassium phosphate pH 7.4, 300 mM NaCl, 0.1 mM EDTA and 5% sucrose) containing 0.03% NP40 and 5 µg/ml Dextran Alexa Fluor 680 to a final concentration of 3 µM protein per zygote.

To stain DNA in live cells, zygotes were washed and imaged in M2 medium containing 250 nM SiR-DNA (Spirochrome, dissolved in DMSO at 1 mM).

### Live-cell confocal and super-resolution microscopy

Images were acquired with Zeiss LSM800 and LSM880 confocal laser-scanning microscopes at 37 °C equipped with a ×40 C-Apochromat 1.2 NA water-immersion objective and controlled by the software ZEN Black 2.1 (LSM880) or ZEN Blue 2.3 (LSM800) with multi-position mode. For live imaging, zygotes were cultured in drops of M2 medium with or without drugs under mineral oil in MatTek-35 mm glass bottom dishes. Super-resolution time-lapse images of zygotes expressing EMTB-mClover or GFP-UtrCH were acquired using the Airyscan module on the Zeiss LSM800 microscope and processed post-acquisition using ZEN Blue.

For pronuclear migration, images were typically acquired at a temporal resolution of 15 min covering an 81 µm range (every 3 µm) with an optical slice thickness of 3 µm for a total duration of 18 h. mScarlet-Rab11a images were acquired covering an 81 µm range (every 1.5 µm) with an optical slice thickness of 2.5 µm. To monitor localization of Rab11a, Spire2 and Formin-2 at the fertilization cap, images were acquired at a temporal resolution of 3 min covering a 20-µm-thick subvolume centred around the pronuclei (every 1.5 µm) with an optical slice thickness of 2 µm for a total duration of 3–4 h. For aMTOC tracking, images were acquired at a temporal resolution of 10 min covering an 81 µm range (every 2.5 µm) with an optical slice thickness of 2 µm for a total duration of 18 h. GFP-UtrCH airyscan images were acquired at a temporal resolution of ~3 or ~30 s at the central plane of the pronucleus. EMTB-mClover airyscan images were acquired at a temporal resolution of either 15 min covering a 40-µm-thick subvolume centred around the middle slice of the cell at 4-µm intervals, or 3 or 5 min covering 3 µm at 1–1.5-µm intervals. Settings of the 488, 561 and 633 nm lasers were similar to verified live imaging conditions for mouse embryos in a previous report^42^ and did not exceed 0.2% laser intensity to minimize light-induced phototoxic stress.

### Laser ablation

Zygotes were imaged in M2 medium containing 250 nM SiR-DNA (Spirochrome, dissolved in DMSO at 1 mM) and 1X CellMask Green (Invitrogen C37608, dissolved in DMSO) at 20-s intervals prior to and after laser ablation. Once male pronuclei had moved roughly 1 µm away from the cell surface, a region of 8 × 0.7 × 7 µm (eight sections at 1 µm) was two-photon laser ablated at 790 nm with 18% laser power. The ablated region was manually defined to perform eight cuts in z using the LSM980 confocal laser-scanning microscope. The ablated regions were either located between cell surface and male pronucleus (behind the male pronucleus) or in the cytoplasm in front of the male pronucleus. After ablation, imaging of zygotes was restarted within 70–100 s.

### Analysis of cytoplasmic streaming

Zygotes and their pronuclei (SiR-DNA) were imaged in 3–10-s intervals. Transmission images were used to detect and analyse cytoplasmic streaming using the ImageJ PIV Plugin by Qingzong Tseng^56^. The following parameters were used: piv1 = 128, sw1 = 256, vs1 = 64, piv2 = 64, sw2 = 128, vs2 = 32, piv3 = 0, sw3 = 0, vs3 = 0. Single z-slices were analyzed using an in-house ImageJ macro to process full timeseries in one go. The results were averaged over 5-min periods to highlight relevant flows using Python 3.7. Plotting of the vectors was conducted in Matlab 2018b due to a bug affecting short vectors in the original plugin. Vectors outside of the cell were removed.

### Immunofluorescence microscopy

Zygotes were fixed at time points indicated in hours post insemination (hpi, start of IVF). We experimentally determined that pronuclear formation occurs 3.9 ± 0.5 hpi (Supplementary Fig. 1l), which was used as a reference time point for immunofluorescence studies to fix zygotes close to the time of pronuclear formation in Supplementary Figs. 1h, 2b, g, i and 4h. Zygotes were treated for 5–10 s in 2.5% Triton X-100 diluted in water before they were fixed for 25 min at 37 °C with 100 mM HEPES, 50 mM EGTA, 10 mM MgSO_4_, 2% formaldehyde and 0.4% Triton x-100 and extracted in PBS supplemented with 0.5% Triton X-100 at 4 °C overnight. Antibody incubation, F-actin and DNA staining were performed in PBS, 3% BSA and 0.5% Triton X-100 for 2–4 h at room temperature, except Rab11a staining was performed in PBS, 12% BSA and 0.5% Triton X-100 overnight at 4 °C. Primary antibodies used in this study: rat anti-α-tubulin (Serotec, MCA78G, 1:500), rabbit anti-Rab11a (Abcam, ab65200, 1:2000) and mouse anti-pericentrin (BD Biosciences, #611815, 1:1000). Alexa Fluor-488-labelled anti-mouse, Alexa Fluor-546-labelled anti-rabbit and Alexa Fluor-647-labelled anti-rat (Molecular Probes, 1:400) were used as secondary antibodies. DNA was stained with 25 µg/ml Hoechst 33342 (Molecular Probes); F-actin was stained with Alexa Fluor-488-phalloidin (Molecular Probes, 1:20). Images were acquired with LSM800 and LSM880 confocal microscopes equipped with a ×40 C-Apochromat 1.2 NA water-immersion objective at room temperature. Immunofluorescence images were typically acquired at an optical slice thickness of 2 µm confocal sections covering a subvolume or the entire oocyte at 1-µm intervals if not indicated otherwise. For visualizing F-actin, single optical sections of F-actin in the equatorial region of zygotes were acquired using the Airyscan module on the Zeiss LSM800 microscope. Images in control and perturbed situations were acquired with identical imaging conditions. Care was taken that images were not saturated during acquisition (except for co-imaging of the meiosis II spindle and the interphase microtubule network).

### Image analysis and statistics

Microscopic images were processed in ZEN Blue 2.3 and ImageJ (version 1.52p). 3D reconstructions and tracking of pronuclei and aMTOCs were performed using Imaris 9.2 and 9.3 (Bitplane). Cells were reconstructed as using the ImarisCell Module based on plasma membrane labelling. Pronuclei were tracked automatically using the Imaris tracking function and verified manually. The distance between cell centre and pronuclei centroids was calculated from the centres of homogenous mass in Microsoft Excel for every time point as illustrated in Supplementary Fig. 1d. Average velocities were obtained by averaging instantaneous velocities within a specified time-interval for each pronucleus. aMTOCs were detected using the Imaris spot detection and tracked manually.

Pronuclear migration was defined as the period from pronuclear formation (time point 0 h) until 15 min prior to onset of chromosome condensation at NEBD. Pronuclear formation, as detected by the emergence of the nuclear envelope labelled by GFP-LaminB1, coincided with the onset of chromosome decondensation characterized by the sudden volume increase of the H2B-mCherry-labelled DNA mass (Supplementary Fig. 1a–c), which was thereafter used as determinant of pronuclear formation. In Supplementary Fig. 1b, c, pronuclear volumes were normalized to the respective volume at the end of pronuclear migration. Plots for pronuclear migration were aligned relative to pronuclear formation, the time we referred to as time 0 h. Images are displayed as single sections for the cell surface (MyrGFP, equatorial plane) and as a maximum intensity projection of the entire cell for chromosomes (H2B-mCherry) in Figs. 1a, 2a, 3a, 5a, 6a, 7b, h, 8a and 9a. Distinct phases of migration for male and female pronuclei were defined based on typical pronuclear migratory behaviour with the following speeds: fast (>0.1 µm/min), medium (0.05–0.1 µm/min) and slow (<0.05 µm) (Fig. 1a–c).

Only zygotes were analyzed whose pronuclei had not yet formed at the start of acquisition and that progressed to the two-cell stage during the first 18 h of acquisition as these were considered healthy, except for in the cases of treatment with cytochalasin D or p150-CC1, which both caused a delay of NEBD. Furthermore, zygotes were excluded that contained more or less than two pronuclei (parthenogenesis, polyspermy) or male pronuclei forming at a distance from the cell surface (in untreated zygotes 3/56 zygotes)and that exhibited cortical deformations after microinjection of SNAP-Rab11a^S25N^, precluding an accurate determination of the centre of the zygote as required for pronuclear tracking (25/65 zygotes).

To confirm reproducibility between repetitions and experiments, mean distances over time from independent repetitions were plotted (Supplementary Fig. 12a–q).

Measurements of average velocities suggested that different manipulations affect different portions of pronuclear migration. To confirm the results of our approach, we also used curve fitting with a piecewise linear function to estimate slopes in different portions of the curve after concatenation of all data points (OriginLab). The function of the piecewise linear function can be expressed as follows:

$$y = \left\{ {\begin{array}{*{20}{c}} {\frac{{{y}_1\left( {{x}_3\, -\, x} \right)\, +\, {y}_3\left( {x\, -\, {x}_1} \right)}}{{{x}_3 - {x}_1}},{\rm{if}}\,x\, <\, x_3} \\ {\frac{{{y}_3\left( {{x}_3\, -\, x} \right)\, +\, {y}_3\left( {x\, -\, {x}_1} \right)}}{{{x}_2\, -\, {x}_3}},{\rm{if}}\,x \ge x_3} \end{array}} \right.$$

To ensure to stay within the time window, in which pronuclei move freely, we performed the fitting between 0 and 5 h, whereby *x*_1_ = 0 and *x*_2_ = 5 were fixed and *x*_3_ is the intersection time point. Results of our analysis are summarized in Supplementary Fig. 13a–q and Supplementary Table 2 and confirm our previous approach.

In Supplementary Fig. 8b, d, microtubules asters/aMTOCs were manually counted. For aMTOC tracking, at least six aMTOCs per zygote were tracked using Imaris from the onset to the end of pronuclear migration. Microtubule density in Supplementary Fig. 7a was measured as an average of four regions per cell over time from a maximum projection after drift correction in Imaris and then normalized to pronuclear formation (0 h). Oil droplets were tracked manually using ImageJ (TrackMate Plugin). Temporal-colour coded images in Fig. 8h and Supplementary Fig. 9h were generated in ImageJ.

Average velocities of pronuclei and aMTOCs towards the cell centre were calculated from instantaneous velocities, which were averaged for each pronucleus/aMTOC within the specified interval and plotted as individual dots in the average velocity-over-time-scatter plots.

In live-cell experiments of zygotes expressing mScarlet-Rab11a, vesicles were counted manually in the lower half of the zygote. In immunofluorescence with anti-Rab11a, vesicles were counted automatically in a segment of 25 × 25 × 16.5 µm^3^ around the equatorial plane of the cell excluding regions overlapping with pronuclear positions using Imaris spot detection (different spot sizes with region growing and an estimated diameter of 0.3 µm, filtered volume of spots > 0.0283 µm^3^). In immunofluorescence, spots were categorized depending on their volume. Spots above 0.5 µm^3^ were clearly discernible from background and considered as vesicles. Spots below 0.5 µm^3^ were difficult to discriminate from background signal and were therefore excluded.

To quantify the density of the cytoplasmic actin network, the mean intensity of Alexa Fluor-488-phalloidin was measured in the cytoplasm (average from 6 to 12 regions per cell) and in a region outside the zygote for background subtraction using ImageJ. The disruption of the actin cortex was determined by eye, and the number of holes in the actin cortex was counted manually (areas of >1 µm in the cortex that were devoid of phalloidin).

Graphs and plots were created using GraphPad Prism 5.0.3 and OriginPro 9.1 (OriginLab). Statistical plots show mean (horizontal middle lines), and standard deviation (whiskers). The box plot shows median (line), mean (small square), 5th, 95th (whiskers) and 25th and 75th percentile (boxes enclosing 50% of the data). Mean, standard deviation and statistical significance based on two-tailed Student’s *t* test (assuming unequal variances) were calculated in GraphPad Prism. *p* values are designated as * (*p* < 0.05), ** (*p* < 0.005) and *** (*p* < 0.0005). Nonsignificant values are indicated as n.s. Experiments were performed at least three times. Figures were assembled in Adobe Illustrator.

### Expression constructs and mRNA synthesis

To generate a membrane-anchored GFP, a sequence encoding for a myristoylation signal (MGCIKSKRKDNLNDDEAA^57^) was inserted N-terminally in frame with mEGFP by PCR using primers forward (GCTAGCATGGGCTGCATCAAGAGCAAGCGCAAGGACAACCTGAACGACGACGAGGCCGCCATGGTGAGCAAGGGCGAGGAGC) and reverse (GAATTCGAAGCTTGAGCTCG) from pmEGFP-C1 (Addgene, #227464) as a template. The NheI-EcoRI flanked fragment was subsequently cloned into pGEMHE to generate pGEMHE-MyrGFP. To generate pGEMHE-mScarlet-Rab11a, mScarlet was first cloned into the NheI-XhoI of pGEMHE by PCR with pmScarlet-C1 (Addgene #85042) as a template. Subsequently, the Rab11a sequence from pGEMHE-SNAP-Rab11 was inserted into the HindIII site via Gibson Assembly (New England Biolabs). pGEMHE-mClover-Spire2 was obtained from pGEMHE-mCherry-Spire2^35^ by switching mCherry with mClover via NheI-XhoI by PCR from pKanCMV-mClover3-18aa-TUBA (gift from Michael Lin, Addgene #74253). To generate pGEMHE-SNAP-Spire2 and pGEMHE-mScarlet-Spire2, SNAPf from pSNAPf (New England Biolabs) or mScarlet was cloned into the NheI-BamHI site of pGEMHE-mClover3-Spire2. For pGEMHE-Fmn2-EGFP, Fmn2-EGFP was cloned into BglII-NotI site of pGEMHE from pCS2-Fmn2-EGFP^58^. For pGEMHE-EGFP-UtrCH, EGFP-UtrCH was cloned into the AgeI-NotI site of pGEMHE from pCS2-EGFP-UtrCH^59^. For pGEMHE-EMTB-mClover containing the microtubule binding domain of MAP7/Ensconsin^60^, the 3xEGFP sequence in pGEMHE-EMTB-3xEGFP (gift from Jan Ellenberg) was switched with the mClover sequence from pTALYM3B15 (Addgene #47878) via BamHI-NotI site. The previously published sequence EGFP-LaminB1^61^ was inserted into pGEMHE to generate pGEMHE-EGFP-LaminB1. For pGEMHE-miRFP670-Trim21, the EGFP sequence in pGEMHE-EGFP-Trim21^46^ was replaced by the miRFP670 sequence from pmiRFP670-N1 (gift from Vladislav Verkhusha, Addgene # 79987) via AgeI-BglII site. Expression constructs pGEMHE-H2B-mCherry, pGEMHE-H2B-mEGFP^46^, pGEMHE-SNAP-Rab11a, pGEMHE-SNAP-Rab11a^S25N^ ^62^, pGEMHE-GFP-Spire2^35^ and pGEMHE-Cep192-mScarlet^63^ were generated in previous studies as indicated. All primers used in this study to generate the above-named plasmids are listed in Supplementary Table 3.

For in vitro mRNA transcription, pGEMHE plasmids were linearized with AscI, NheI or SphI and capped mRNA was synthesized with T7 polymerase (Ambion mMessage mMachine T7 Kit) according to manufacturer instructions. mRNA concentrations were determined using NanoDrop.

### Protein expression and purification of His-tagged proteins

All constructs were expressed in *E. coli* BL21-CodonPlus (DE3) (Agilent Technologies #230245) from pET-28a with a C-terminal His-tag. pET-28a-p150-CC1-His (gift from Jan Ellenberg) was generated in a previous study^44^. To generate pET-28a-FH2-His and as a negative control pET-28a-MBP-His, p150-CC1 was replaced by the coding sequences of FH2 and MBP amplified from pGEX-6P1-FH2 (gift from Marie France-Carlier^40^) and pET-21a-MBP-His (Addgene # 38006) and inserted via XbaI-XhoI.

For p150-CC1-6xHis purification, bacterial cultures were induced with 1 mM isopropyl-β-d-thiogalactoside at 37 °C overnight. Bacteria were lysed in binding buffer (50 mM NaPO_4_ buffer, pH 8.0; 300 mM NaCl; 2 mM β-mercaptoethanol; 15% glycerol) by sonication. p150-CC1-6xHis and MBP-6xHis were bound to Ni-NTA resin (Qiagen) and eluted with 250 mM imidazole in binding buffer. Buffer exchange to PBS was carried out using Slide-A-Lyzer Dialysis Cassettes (ThermoFisher Scientific). Proteins were concentrated using Amicon Ultra centrifugal filter units (Millipore) at 14,000 × *g*, aliquoted, flash frozen in liquid nitrogen and stored at −80 °C. All purification was carried out at 4 °C to minimize protein aggregation. Proteins were analyzed by SDS-polyacrylamide gel electrophoresis and Coomassie staining (Biorad) and protein concentration was determined using BSA (NEB) as a reference (Supplementary Fig. 7c, d).

FH2-6xHis and MBP-6xHis were purified using Ni-NTA as described for p150-CC1-6xHis, but with the following adaptations: bacterial cultures were induced with 1 mM isopropyl-β-d-thiogalactoside at 16 °C overnight. Bacteria were lysed in binding buffer (20 mM KPO_4_ buffer, pH 7.4; 30 mM NaCl, 3 mM DTT, 5% sucrose and 0.1 mM EDTA). After elution, buffer was exchanged to binding buffer without DTT (Supplementary Fig. 6a, b).

A presentation of the uncropped full scan gels is presented in the Source Data file provided with this paper.

### Reporting summary

Further information on research design is available in the Nature Research Reporting Summary linked to this article.

## Supplementary information

Supplementary Information

Description of Additional Supplementary Files

Supplementary Movie 1

Supplementary Movie 2

Supplementary Movie 3

Supplementary Movie 4

Supplementary Movie 5

Supplementary Movie 6

Supplementary Movie 7

Supplementary Movie 8

Supplementary Movie 9

Supplementary Movie 10

Supplementary Movie 11

Supplementary Movie 12

Supplementary Movie 13

Supplementary Movie 14

Supplementary Movie 15

Supplementary Movie 16

Supplementary Movie 17

Supplementary Movie 18

Supplementary Movie 19

Supplementary Movie 20

Reporting Summary

## Data Availability

All relevant and raw data supporting the finding of this study are available from the corresponding author upon request for the following figures: Figs. 1b, 2c, 3b, c, 5c, 6b, c, 7c, d, i, j, 8b, c, i, S1f, j, S3b, c, g, j, S5c, e, S6c, e, S7a, g, i, S8b, e, f, S9b, c, i, S10a, b. The primary microscopy data were not uploaded to a data repository due to their large size, but are available from the authors upon request. Source data are provided with this paper.

## Source data

Source Data
