# Supplementary material for: Two mechanisms drive pronuclear migration in mouse zygotes
Source: Nat Commun. 2021 Feb 5;12:841. doi: 10.1038/s41467-021-21020-x (PMC7864974; doi:10.1038/s41467-021-21020-x)
Supplement: Supplementary file 2 — Description of Additional Supplementary Files [file 41467_2021_21020_MOESM2_ESM.pdf]

Description of Additional Supplementary Files

File Name: Supplementary Movie 1
Description: Pronuclear formation (0 h) and pronuclear migration in a zygote expressing H2B-mCherry (DNA, magenta) and MyrGFP (cell surface, white).

File Name: Supplementary Movie 2
Description: Pronuclear migration in zygotes expressing H2B-mCherry (DNA, magenta) and MyrGFP (cell surface, white) and treated with DMSO (control, top) or 1 µM Nocodazole relative to pronuclear formation (0 h).

File Name: Supplementary Movie 3
Description: Pronuclear migration in zygotes expressing H2B-mCherry (DNA, magenta) and MyrGFP (cell surface, white) together with SNAP-Rab11a (control, top) or SNAP-Rab11aS25N (bottom) relative to pronuclear formation (0 h).

File Name: Supplementary Movie 4
Description: Oil droplet motion in vicinity of the male pronucleus in a zygote expressing H2B-mCherry (DNA, magenta) relative to pronuclear formation (0 min).

File Name: Supplementary Movie 5
Description: Cortical Rab11a enrichment behind the male pronucleus in a zygote expressing mScarlet-Rab11a (white) and H2B-eGFP (DNA, magenta) relative to pronuclear formation (0 min).
File Name: Supplementary Movie 6
Description: Cortical Spire2 enrichment behind the male pronucleus in a zygote expressing mClover3-Spire2 (green) and H2B-mCherry (DNA, magenta) relative to pronuclear formation (0 min).
File Name: Supplementary Movie 7
Description: Cortical F-actin enrichment behind male pronucleus in a zygote expressing eGFP-UtrCH (pseudocolour) represented in fluorescence intensity map.

File Name: Supplementary Movie 8
Description: Cortical F-actin enrichment behind male pronucleus in a zygote expressing eGFP-UtrCH (pseudocolour) and mScarlet-Spire2 (not shown) represented in fluorescence intensity map.

File Name: Supplementary Movie 9
Description: Pronuclear migration in zygotes expressing H2B-mCherry (DNA, magenta) and MyrGFP (cell surface, white) upon overexpression of SNAP (control, top) or SNAP-Spire2 (bottom) relative to pronuclear formation (0 h).

File Name: Supplementary Movie 10
Description: Overshooting of pronuclei in zygotes expressing H2B-mCherry (magenta) upon overexpression of EGFP-Spire2 (white) relative to pronuclear formation (0 h) represented as a maximum intensity projection (13 slices, every 5 µm).

File Name: Supplementary Movie 11
Description: Overshooting and reversion of migration of pronuclei in zygotes expressing H2B-mCherry (magenta) upon overexpression of EGFP-Spire2 (white) relative to pronuclear formation (0 h) represented as a maximum intensity projection (13 slices, every 5 µm).

File Name: Supplementary Movie 12
Description: Oil droplet motion in zygotes expressing H2B-mCherry (magenta) upon overexpression of EGFP-Spire2 (green) relative to pronuclear formation (0 h) represented as a maximum intensity projection (13 slices, every 5 µm).

File Name: Supplementary Movie 13
Description: Cortical F-actin enrichment behind female pronucleus in zygote expressing EGFP-UtrCH (pseudocolour) and mScarlet-Spire2 (not shown) and treated with DMSO (control, left) or 1 µM Nocodazole (right) represented in fluorescence intensity map.

File Name: Supplementary Movie 14
Description: Formation of the microtubule network during pronuclear stage in zygotes expressing H2B-mCherry (DNA, magenta) and EMTB-mClover (microtubules, white) relative to pronuclear formation (0 h).

File Name: Supplementary Movie 15
Description: Pronuclear migration in zygotes expressing H2B-mCherry (DNA, magenta) and MyrGFP (cell surface, white) treated with DMSO (control, top) or 10 µM Nocodazole (bottom) relative to pronuclear formation (0 h).

File Name: Supplementary Movie 16
Description: The microtubule network during pronuclear stage in zygotes expressing EMTB-mClover (microtubules, white) and injected with 30uM MBP (control, top) or p150-CC1 (bottom)

File Name: Supplementary Movie 17
Description: Microtubules contact cell cortex during pronuclear stage in zygotes expressing EMTB-mClover (microtubules, white).

File Name: Supplementary Movie 18
Description: Microtubule originating from aMTOCs contact cell cortex in mouse zygotes expressing EMTB-mClover (microtubules, white).

File Name: Supplementary Movie 19
Description: The microtubule network during pronuclear stage in zygotes expressing H2B-mCherry (DNA, magenta) and EMTB-mClover (microtubules, white) and treated with DMSO (Control, top) or 5 µg/mL Cytochalasin D (middle and bottom) relative to pronuclear formation (0 h).

File Name: Supplementary Movie 20
Description: Pronuclear migration in zygotes expressing H2B-mCherry (DNA, magenta) and MyrGFP (cell surface, white) together with SNAP-Rab11a or SNAP-Rab11aS25N (bottom) and treated with DMSO (control, top) or 10 µM Nocodazole (bottom) relative to pronuclear formation (0 h).
