# Supplementary material for: Two mechanisms drive pronuclear migration in mouse zygotes
Source: Nat Commun. 2021 Feb 5;12:841. doi: 10.1038/s41467-021-21020-x (PMC7864974; doi:10.1038/s41467-021-21020-x)
Supplement: Supplementary file 23 — Reporting Summary [file 41467_2021_21020_MOESM23_ESM.pdf]

nature research

Corresponding author(s): Dr. Melina Schuh
Last updated by author(s): 13/12/2020

Reporting Summary

Nature Research wishes to improve the reproducibility of the work that we publish. This form provides structure for consistency and transparency in reporting. For further information on Nature Research policies, see our Editorial Policies and the Editorial Policy Checklist.

Statistics

For all statistical analyses, confirm that the following items are present in the figure legend, table legend, main text, or Methods section.

n/a	Confirmed	
☐	☒	The exact sample size (n) for each experimental group/condition, given as a discrete number and unit of measurement
☐	☒	A statement on whether measurements were taken from distinct samples or whether the same sample was measured repeatedly
☐	☒	The statistical test(s) used AND whether they are one- or two-sided<br>*Only common tests should be described solely by name; describe more complex techniques in the Methods section.*
☒	☐	A description of all covariates tested
☒	☐	A description of any assumptions or corrections, such as tests of normality and adjustment for multiple comparisons
☐	☒	A full description of the statistical parameters including central tendency (e.g. means) or other basic estimates (e.g. regression coefficient) AND variation (e.g. standard deviation) or associated estimates of uncertainty (e.g. confidence intervals)
☐	☒	For null hypothesis testing, the test statistic (e.g. F, t, r) with confidence intervals, effect sizes, degrees of freedom and P value noted<br>*Give P values as exact values whenever suitable.*
☒	☐	For Bayesian analysis, information on the choice of priors and Markov chain Monte Carlo settings
☒	☐	For hierarchical and complex designs, identification of the appropriate level for tests and full reporting of outcomes
☒	☐	Estimates of effect sizes (e.g. Cohen's d, Pearson's r), indicating how they were calculated

Our web collection on statistics for biologists contains articles on many of the points above.

Software and code

Policy information about availability of computer code

Data collection	ZEN Black 2.1 and ZEN Blue 2.3
Data analysis	Imaris 9.2 and 9.3, Excel 2016, Graph Pad Prism 5.0.3., Origin 9.1, Image J 1.52p, Phyton 3.7, Matlab 2018b

For manuscripts utilizing custom algorithms or software that are central to the research but not yet described in published literature, software must be made available to editors and reviewers. We strongly encourage code deposition in a community repository (e.g. GitHub). See the Nature Research guidelines for submitting code & software for further information.

Data

Policy information about availability of data

All manuscripts must include a data availability statement. This statement should provide the following information, where applicable:

- Accession codes, unique identifiers, or web links for publicly available datasets
- A list of figures that have associated raw data
- A description of any restrictions on data availability

All relevant and raw data supporting the finding of this study are available from the corresponding author upon request for following figures: Fig. 1b, 2c, 3b, c, 5c, 6b, c, 7c, d, i, j, 8b, c, i, S1f, j, S3b, c, g, j, S5c, e, S6c, e, S7a, g, i, S8b, e, f, S9b, c, i, S10a, b. The primary microscopy data were not uploaded to a data repository due to their large size, but are available from the authors upon request. Source data (for Figs. 1b, c, 2b-d, 3b-f, 4e, 5c, d, f, g, 6b-f, 7c-g, i-m, 8b-e, i, 9b-d, Supplementary Figs. 1b, c, e-g, i-n, 2c-f, h, j, 3b-e, g, j, k, 4a-c, e, i, k, l, 5a, e, 6a-e, 7a, c, d, f-i, k, 8b, d-h, 9b-g, i, 10a, b, 11a-e, 12a-o) are provided as a Source Data file with this paper. A reporting summary for this article is available as a supplementary information file.

Field-specific reporting

Please select the one below that is the best fit for your research. If you are not sure, read the appropriate sections before making your selection.

☒ Life sciences ☐ Behavioural & social sciences ☐ Ecological, evolutionary & environmental sciences

For a reference copy of the document with all sections, see nature.com/documents/nr-reporting-summary-flat.pdf

Life sciences study design

All studies must disclose on these points even when the disclosure is negative.

Sample size	No sample size calculations were performed. In retrospective, achieved sample sizes were determined to be adequate based on the magnitude and consistency of measurable differences between groups.<br>Most importantly, sample size per experiment was dictated by the number of MII eggs that could be processed (microinjection, in vitro fertilization and live imaging) within a reasonable time by one researcher without greatly affecting egg and embryo quality. Following parameters further dictated final sample size per experiment, which varied on different experimental days:<br>1. Number of MII eggs obtained from 2 supervulated females<br>2. Survival rate of eggs and zygotes after microinjection<br>3. Fertilization rate
Data exclusions	Following zygotes were excluded from analysis: 1. Zygotes whose pronuclei formed before the start of acquisition as early pronuclear migration events were missed in these zygotes. 2. Zygotes who had not progressed to the two-cell stage during the 18 hours of acquisition as these were considered abnormal based on a previous studies. 3. Zygotes with other aberrant development defects, e.g. more than two pronuclei (polyspermy, dispersion of maternal chromosomes). 4. Zygotes with male pronuclei (3/56 zygotes) forming at a distance from the cell surface as these did not show male-specific migration behaviour and were not representative for male pronuclear migration. 5. Zygotes undergoing abnormal cortical deformations after microinjection of SNAP-Rab11aS25N as the distance of pronuclei to cell centre could not be determined accurately.<br>These exclusion criteria were not pre-established, but identified after experiments were performed.
Replication	Experiments were repeated between 3 to 6 times, the exact number of replicates are indicated for each data set in the associated figure legends. Replicate experiments were similar (Supplementary Fig. 12), and all attempts at replication were successful.
Randomization	Oocytes were collected from typically 2 mice per experiment and randomly distributed between control and tested conditions.
Blinding	Investigators were not blinded during experiments and data analysis nor group allocation during data collection, as each experiment was performed typically by one researcher alone.<br>Thus, blinding during group allocation was not possible to ensure samples received the right treatment/manipulation during experiment. Blinding was not performed during data analysis, as data collection and data analysis was performed by the same researcher.

Reporting for specific materials, systems and methods

We require information from authors about some types of materials, experimental systems and methods used in many studies. Here, indicate whether each material, system or method listed is relevant to your study. If you are not sure if a list item applies to your research, read the appropriate section before selecting a response.

Materials & experimental systems

n/a	Involved in the study	
☐	☒	Antibodies
☒	☐	Eukaryotic cell lines
☒	☐	Palaeontology and archaeology
☐	☒	Animals and other organisms
☒	☐	Human research participants
☒	☐	Clinical data
☒	☐	Dual use research of concern

Methods

n/a	Involved in the study	
☒	☐	ChIP-seq
☒	☐	Flow cytometry
☒	☐	MRI-based neuroimaging

Antibodies

Antibodies used	Primary antibodies used in this study: rat anti-α-tubulin (Serotec, MCA78G, 1:500), rabbit anti-Rab11a (Abcam, ab65200, 1:2000), mouse anti-Pericentrin (BD Biosciences, #611815, 1:1000). Alexa Fluor-488-labelled anti-mouse, Alexa Fluor-546-labelled anti-rabbit and Alexa Fluor-647-labelled anti-rat (Molecular Probes, 1:400) were used as secondary antibodies. DNA was stained with 25 µg/ml Hoechst 33342 (Molecular Probes); F-actin was stained with Alexa Fluor-488-phalloidin (Molecular Probes, 1:20).<br>For Trim-away, mouse IgG (Sigma, #12-371) and mouse anti-Pericentrin (BD Biosciences, #611815) were used.

Validation	All used antibodies were validated commercially. Certificates of analysis for the approved applications by the manufacturer and references are available on the company websites.<br>- rat anti-α-tubulin: https://www.bio-rad-antibodies.com/monoclonal/yeast-tubulin-alpha-antibody-yol1-34-mca78.html?f=purified<br>- rabbit anti-Rab11a: https://www.abcam.com/rab11a-antibody-ab65200.html<br>- mouse anti-Pericentrin: https://www.bdbiosciences.com/us/reagents/research/antibodies-buffers/cell-biology-reagents/cell-biology-antibodies/purified-mouse-anti-mouse-pericentrin-30pericentrin/p/611814

Animals and other organisms

Policy information about studies involving animals; ARRIVE guidelines recommended for reporting animal research

Laboratory animals	Mice of following strains were used: 8-12 weeks old C57BL/6J x CBA/OlaHsd F1 females and > 12 weeks old C57BL/6J x CBA/OlaHsd F1 males. Housing conditions for the mice were as follows: 21 °C ambient temperature, 52-55% humidity and with a 14-hour light/10-hour dark cycle.
Wild animals	Study did not involve wild animals.
Field-collected samples	Study did not involve samples collected from the field.
Ethics oversight	The maintenance and handling of all mice were performed in the MPI-BPC animal facility according to international animal welfare rules (Federation for Laboratory Animal Science Associations guidelines and recommendations). Requirements of formal control of the German national authorities, and the study received approval by the Niedersächsisches Landesamt für Verbraucherschutz und Lebensmittelsicherheit (LAVES).

Note that full information on the approval of the study protocol must also be provided in the manuscript.
