# Supplementary material for: Two mechanisms drive pronuclear migration in mouse zygotes
Source: Nat Commun. 2021 Feb 5;12:841. doi: 10.1038/s41467-021-21020-x (PMC7864974; doi:10.1038/s41467-021-21020-x)
Supplement: Supplementary file 1 — Supplementary Information [file 41467_2021_21020_MOESM1_ESM.pdf]

Supplementary Materials

Supplementary Figures 1-13

Supplementary Tables 1-3

Supplementary Figure 1

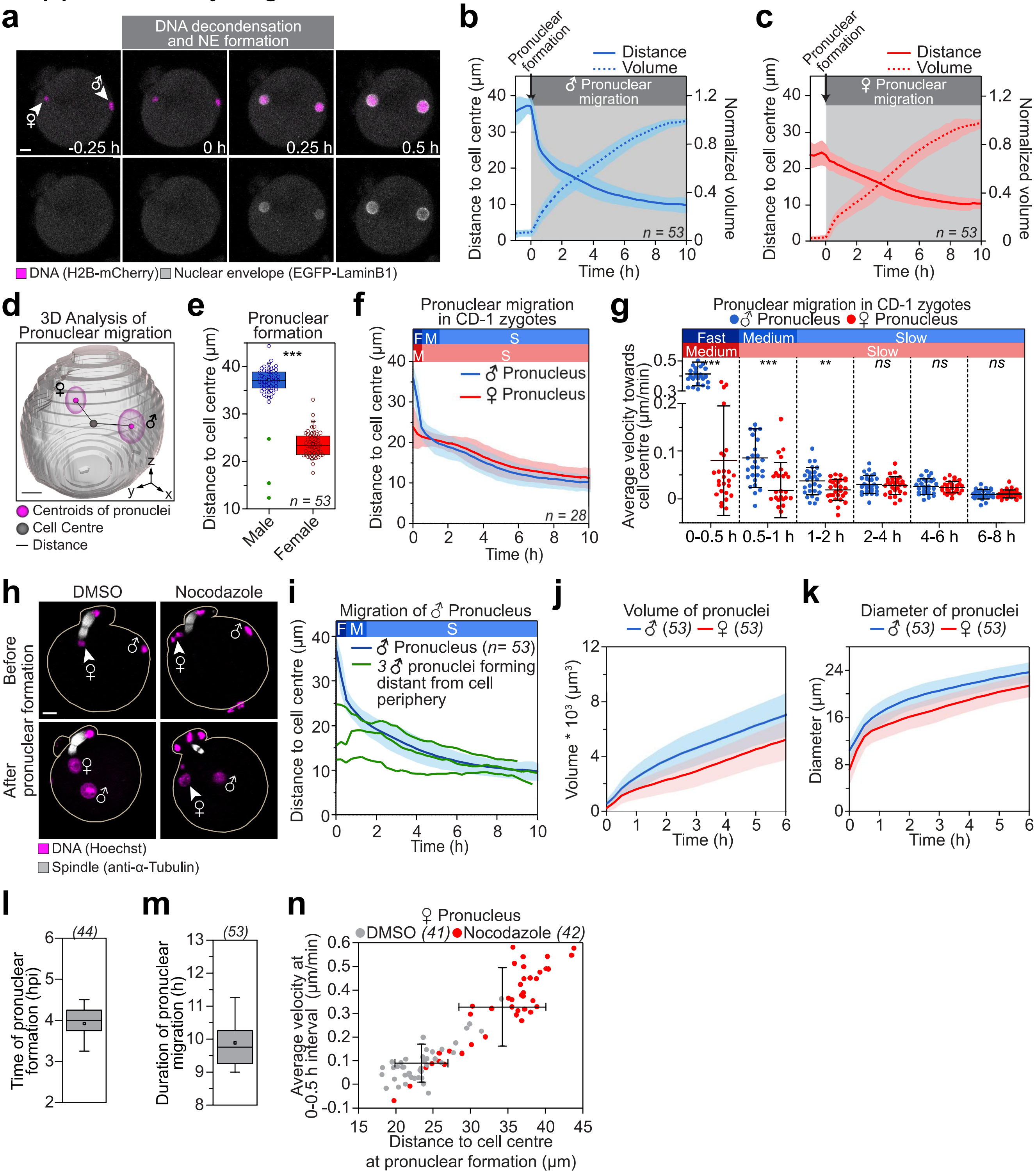

Supplementary Figure 1

(a) Three-dimensional time-lapse images of pronuclei (H2B-mCherry) and the nuclear envelope (NE, EGFP-LaminB1) relative to pronuclear formation (0 h) in live zygotes (z-projection of 5 sections, every 3 µm). Female (♀) and male (♂) genomes are labelled.

(b, c) The mean distance to cell centre (thick line) and normalized volume of male (♂, (b)) and female (♀, (c)) pronuclei relative to pronuclear formation (0 h) was calculated from Fig.1a. The total number of analysed zygotes (italics) was pooled from five independent experiments. S.d. shown as shaded areas. The grey-shaded areas mark the period of pronuclear migration, which is initiated upon pronuclear formation (0 h; black arrow).

(d) Scheme illustrating three-dimensional analysis of pronuclear migration. Surface reconstruction of pronuclei (magenta) and the cell (grey) in Imaris. The distances between the cell centre (centre of homogenous mass) and pronuclei centroids was determined over time starting from pronuclear formation.

(e) Distance of pronuclei centroids to zygote's centre at pronuclear formation in zygotes calculated from Fig. 1a. All individual values (circles) are overlaid with a box plot showing median (line), mean (small square), 5th, 95th (whiskers) and 25th and 75th percentile (boxes enclosing 50% of the data) calculated from the total number of zygotes (italics) and pooled from five independent experiments. Hollow blue dots represent male pronuclei forming beneath the cell surface. Filled green dots represent male pronuclei forming deep inside the cell and were excluded from data analysis (see (i)). Two-tailed student's t-test was used to test for significance ($p<0.0001$).

(f) The mean distance (thick line) of male (♂, blue) and female (♀, red) pronuclei centroids to zygote centre relative to pronuclear formation (0h) was calculated in zygotes from CD-1 female mice. Phases of respective movement speeds are classified as in Fig. 1a. The total number of analysed zygotes (italics) was pooled from three independent experiments. S.d. shown as shaded areas.

(g) Statistical plots of average velocities of male (♂, blue) and female (♀, red) pronuclei during pronuclear migration in zygotes from CD-1 females calculated from (f) with the same sample size as in (f). Statistical plots show mean ± s.d. Two-tailed student's t-test was used to test for significance (from left to right: $p<0.0001$, $p<0.0001$, $p=0.009$, $p=0.6$, $p=0.8$ and $p=0.8$).

(h) Three-dimensional images of zygotes treated with DMSO or 1 µM Nocodazole, fixed 3 and 4.5 hpi and stained for microtubules (anti-α-tubulin) and DNA (Hoechst) (z-projection of 30 section, every 1 µm). The white lines indicate cell outlines.

(i) The mean distance (thick line) of male (♂, blue) pronuclei centroids to zygote centre relative to pronuclear formation (0 h) as shown in Fig. 1b together with three individuals that formed distant from zygote surface. Such events were very rarely observed (3/56 of zygotes from four experiments) and excluded from data analysis, as they do not represent typical behaviour of male pronuclei. Phases of respective movement speeds are classified as in Fig. 1a. S.d. shown as shaded areas.

(j) Volumes (absolute values) of male and female pronuclei from pronuclear formation (0 hours) as obtained from 3D reconstruction as shown in Fig. 1a and plotted in (b) and (c).

(k) Diameters were calculated from volumes from data sets shown in (j). As an approximation, pronuclei were assumed as spheres.

(l) Timepoint of pronuclear formation in hours post insemination (hpi) was calculated from (a). Box plot showing median (line), mean (small square), 5th, 95th (whiskers) and 25th and 75th percentile (box) was calculated from the total number of zygotes (italics) and pooled from three independent experiments.

(m) Duration of pronuclear migration spanning the time from pronuclear formation until onset of mitosis is calculated from Fig. 1a. Box plot showing median (line), mean (small square), 5th, 95th (whiskers) and 25th and 75th percentile (box) was calculated from the total number of analysed zygotes (italics) and pooled from five independent experiments.

(n) Scatter plot showing the average velocity during the first time interval (0-0.5 h) of pronuclear migration over the distance of pronuclei centroids to zygote's centre for female pronuclei after DMSO or Nocodazole treatment from Fig. 2c. Mean and s.d. were calculated and plotted along both axes. Scale bars, 10 µm.

Supplementary Figure 2

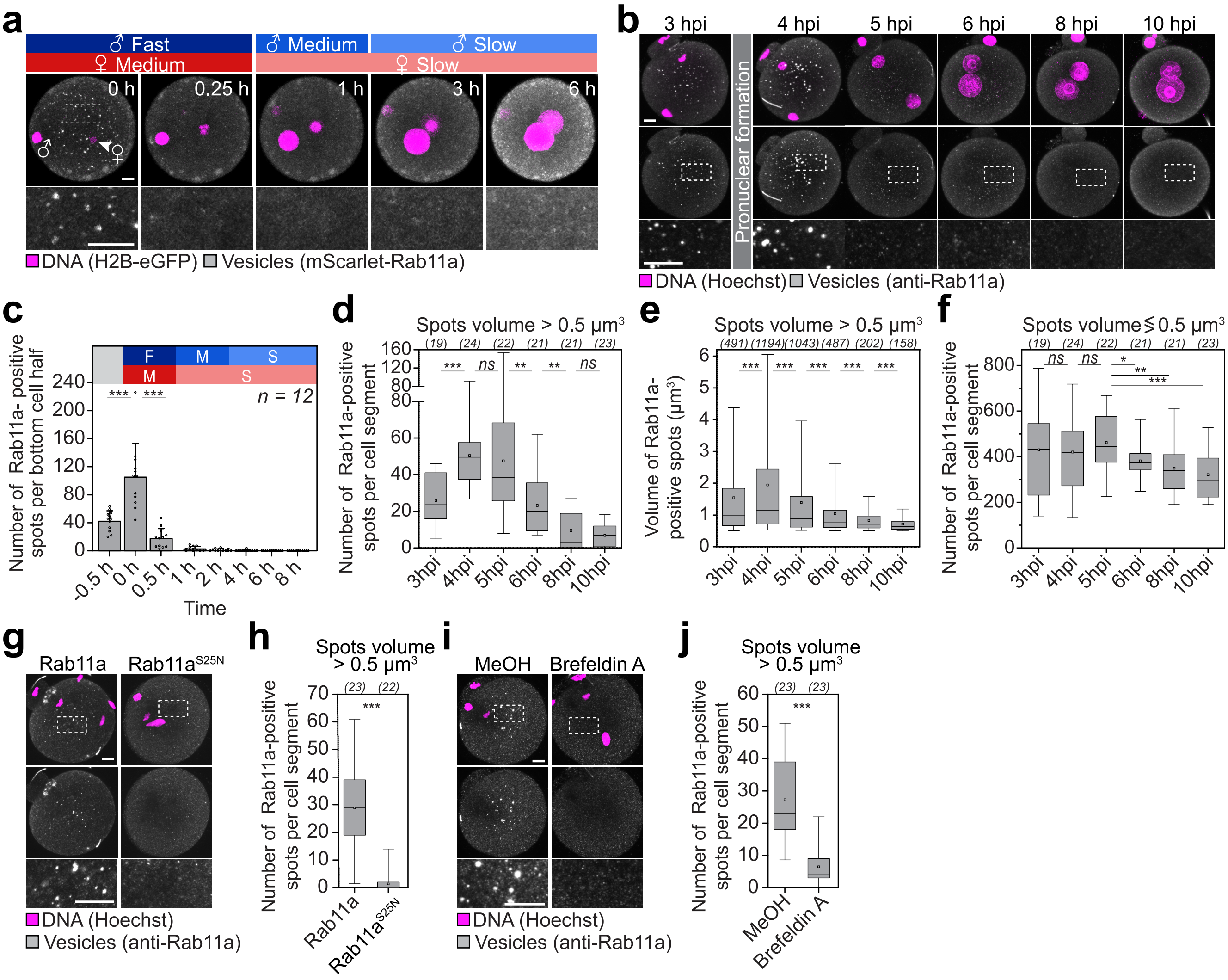

Supplementary Figure 2

(a) Three-dimensional time-lapse images of mScarlet-Rab11a and pronuclei (H2B-EGFP) (z-projection of 11 (Rab11a) or 63 (H2B) sections, every 1.3 µm) relative to pronuclear formation (0 h) in live zygotes. Female (♀) and male (♂) genomes are labelled. Phases of respective movement speeds are classified as in Fig. 1A. The region outlined in the overview is magnified in bottom panel.

(b) Three-dimensional immunofluorescence images of vesicles (anti-Rab11a) and pronuclei (Hoechst) (maximum intensity projection of 11 (Rab11a) or 56 (Hoechst) sections, every 1.5 µm) in zygotes fixed at indicates hours post insemination (hpi). The regions outlined in the overviews are magnified in bottom panel.

(c) Histogram (mean ± s.d.) of number of Rab11a-positive spots (vesicles) per lower zygote half quantified from data sets as shown in (a) from the total number of analysed zygotes (italics) and pooled from three independent experiments. Two-tailed student's t-test was used to test for significance (left to right: p=0.0002 and p<0.0001).

(d) Number of Rab11a-positive spots in a defined cell segment were quantified from data sets as shown in (b). All spots with a volume of 0.5 µm^3 and larger were counted. Box plot showing median (line), mean (small square), 5th, 95th (whiskers) and 25th and 75th percentile (box) was calculated from the total number of analysed zygotes (italics) pooled from three independent experiments. Two-tailed student's t-test was used to test for significance (left to right: p<0.0001, p=0.7, p=0.005, p=0.002 and p=0.3).

(e) Box plot representing the volume of Rab11a-positive vesicles identified in (d) showing median (line), mean (small square), 5th, 95th (whiskers) and 25th and 75th percentile (box) with the same sample size as (d). Two-tailed student's t-test was used to test for significance (left to right: p<0.0001, p<0.0001, p<0.0001, p<0.0001 and p=0.0004).

(f) Box plot representing the number of Rab11a-positive spots in a defined cell segment quantified from data sets as shown in (b). All spots (including spots with a volume below and above 0.5 µm^3) were counted. While larger spots (> 0.5 µm^3) are visible by eye and considered vesicles, smaller spots cannot be detected by eye and considered unspecific background staining. Box plot showing median (line), mean (small square), 5th, 95th (whiskers) and 25th and 75th percentile (box) was calculated from the total number of analysed zygotes (italics) pooled from three independent experiments. Two-tailed student's t-test was used to test for significance (left to right: p=0.9, p=0.4, p=0.03, p=0.008 and p=0.0005).

(g) Three-dimensional images of vesicles (anti-Rab11a) and pronuclei (Hoechst) in immunofluorescence in zygotes expressing SNAP-Rab11a or SNAP-Rab11a^{S25N} and fixed at 3 hpi. The regions outlined in the overviews are magnified in bottom panel.

(h) Box plot representing the number of Rab11a-positive spots in a defined cell segment in zygotes expressing SNAP-Rab11a or SNAP-Rab11a^{S25N} quantified from data sets as shown in g. All spots with a volume of 0.5 µm^3 and larger were counted. Box plot showing median (line), mean (small square), 5th, 95th (whiskers) and 25th and 75th percentile (box) was calculated from a total number of analysed zygotes (italics) pooled from three independent experiments. Two-tailed student's t-test was used to test for significance ($p<0.0001$).

(i) Three-dimensional images of vesicles (anti-Rab11a) and pronuclei (Hoechst) in immunofluorescence in zygotes treated with Methanol (MeOH) or Brefeldin A and fixed at 3 hpi. The regions outlined in the overviews are magnified in bottom panel.

(j) Box plot representing the number of Rab11a-positive spots in a defined cell segment in zygotes treated with MeOH or Brefeldin A were quantified from data sets as shown in (i). All spots with a volume of 0.5 µm^3 and larger were counted. Box plot showing median (line), mean (small square), 5th, 95th (whiskers) and 25th and 75th percentile (box) was calculated from a total number of analysed zygotes (italics) pooled from three independent experiments. Two-tailed student's t-test was used to test for significance ($p<0.0001$). Scale bars, 10 µm.

Supplementary Figure 3

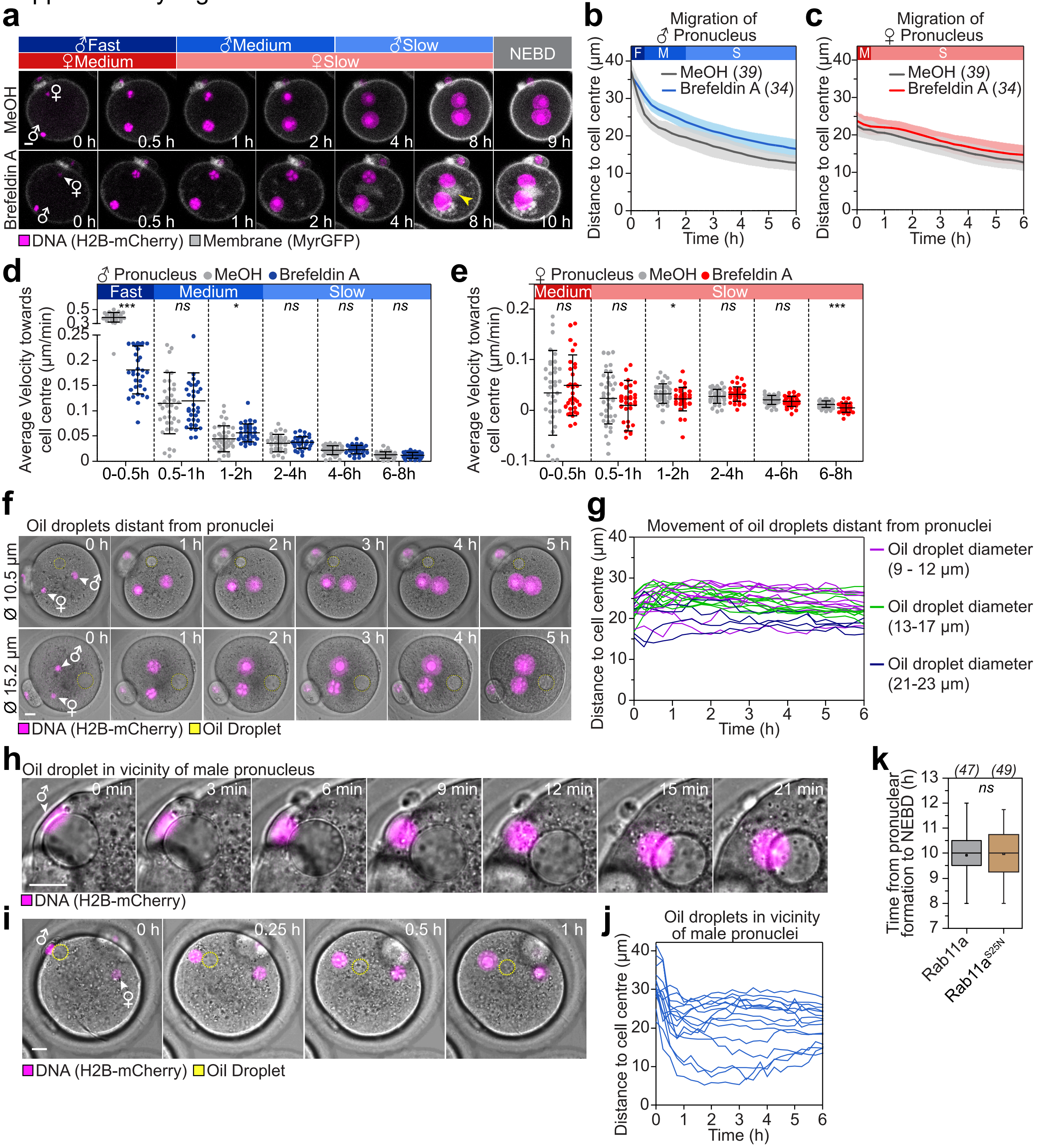

Supplementary Figure 3

(a) Three-dimensional time-lapse images of pronuclei (H2B-mCherry) and the cell surface (MyrGFP) relative to pronuclear formation (0 h) in Methanol (MeOH)- or Brefeldin A-treated zygotes until nuclear envelope breakdown (NEBD). Female (♀) and male (♂) genomes are labelled. Phases of respective movement speeds are classified as in Fig. 1A. The yellow arrowhead marks the increasing accumulation of membranous material in the cell centre, which may act as a barrier for pronuclei to reach cell centre.

(b, c) The mean distance (thick line) of male (♂, (b)) and female (♀, (c)) pronuclei centroids to zygote centre relative to pronuclear formation (0 h) in MeOH- or Brefeldin A-treated zygotes was calculated from data sets as shown in (a) from a total number of analysed zygotes (italics) pooled from three independent experiments. S.d. shown as shaded areas.

(d, e) Statistical plots of average velocities of male (d) and female (e) pronuclei during pronuclear migration in in MeOH- or Brefeldin A-treated zygotes calculated from (b) and (c) with the same sample size as in (b) and (c) respectively. Statistical plots show mean ± s.d. Two-tailed student's t-test was used to test for significance (from left to right: (d) $p<0.0001$, $p=0.7$, $p=0.02$, $p=0.7$, $p=0.5$ and $p=0.4$; (e) $p=0.4$, $p=0.2$, $p=0.04$, $p=0.2$, $p=0.2$ and $p=0.0002$).

(f) Three-dimensional time-lapse images of pronuclei (H2B-mCherry) and oil droplets (yellow circles, introduced into the cell upon microinjection) of different sizes (diameter, Ø) relative to pronuclear formation (0 h) in live zygotes (bright-field).

(g) The mean distance of oil droplet centroids of specified sizes to zygote centre relative to pronuclear formation (0 h) was calculated from data sets as shown in (f).

(h) Three-dimensional time-lapse images of male pronucleus (H2B-mCherry) and oil droplet located in the fertilization cone at pronuclear formation (0 h) in live zygotes (bright-field).

(i) Three-dimensional time-lapse images of pronuclei (H2B-mCherry) and oil droplet in vicinity of the forming male pronucleus (yellow circle) relative to pronuclear formation (0 h) in live zygotes (bright-field).

(j) The mean distance of oil droplet centroids that were in vicinity of male pronuclei during early pronuclear migration to zygote centre relative to pronuclear formation (0 h) was calculated from data sets as shown in (i).

(k) Box plot representing the time from pronuclear formation until NEBD in zygotes expressing SNAP-Rab11a or SNAP-Rab11a^{S25N} quantified from data sets shown in Fig. 2a. Box plot showing median (line), mean (small square), 5th, 95th (whiskers) and 25th and 75th percentile (box) was calculated from a total number of analysed zygotes (italics) pooled from five independent experiments. Two-tailed student’s t-test was used to test for significance ($p=0.8$). Scale bars, 10 µm.

Supplementary Figure 4

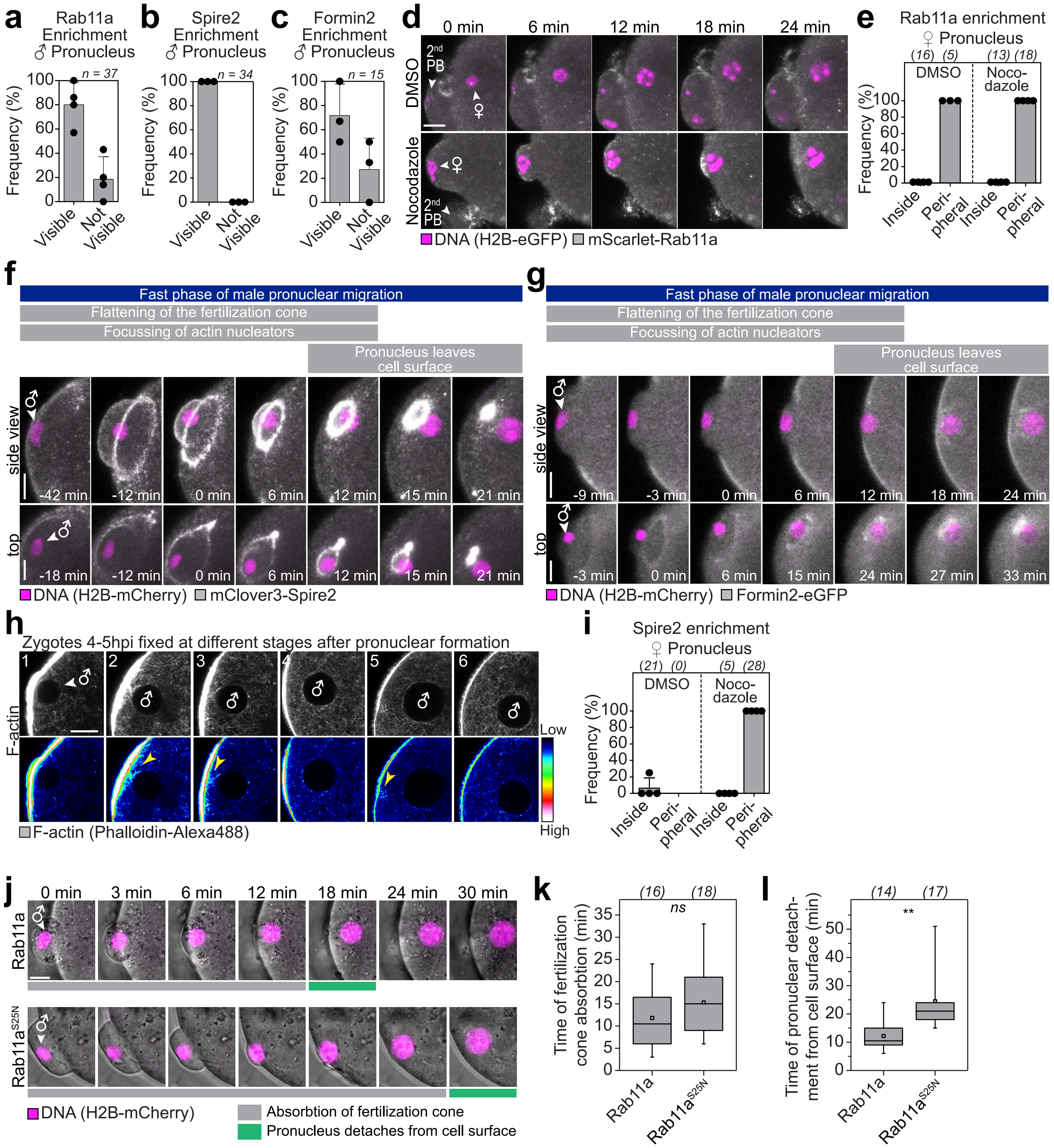

Supplementary Figure 4

(a) Histogram of percentage of zygotes with cortical Rab11a enrichment behind the male (♂) pronuclei as quantified from Fig. 4a. Data are mean, with error bars displaying s.d. from four independent experiments with a total number of analysed zygotes (italics).

(b) Histogram of percentage of zygotes with cortical Spire2 enrichment behind the male (♂) pronuclei as quantified from Fig. 4b. Data are mean, with error bars displaying s.d. from four independent experiments with a total number of analysed zygotes (italics).

(c) Histogram of percentage of zygotes with cortical Formin-2 enrichment behind the male (♂) pronuclei as quantified from Fig. 4c. Data are mean, with error bars displaying s.d. from three independent experiments with a total number of analysed zygotes (italics).

(d) Three-dimensional time-lapse images of cortical mScarlet-Rab11a and female pronuclei (♂, H2B-mCherry) in DMSO- or Nocodazole-treated zygotes relative to pronuclear formation (0 min) in live zygotes (z-projection of 7 sections, every 1.5 µm). Second polar bodies (2nd PB) are labelled.

(e) Histogram illustrating percentage of DMSO- or Nocodazole-treated zygotes with cortical mScarlet-Rab11a enrichment in respect to the position of the forming female pronucleus quantified from (d). Peripheral refers to pronuclei forming close to the zygote's surface, while inside refers to pronuclei forming distant from zygote's surface. Data are mean, with error bars displaying s.d. from three independent experiments from a total number of analysed zygotes (italics).

(f, g) Three-dimensional time-lapse images of mClover3-Spire2 (f) or Formin-2-EGFP (g) and the male pronucleus (♂, H2B-mCherry) relative to pronuclear formation (0 min) in live zygotes (z-projection of 20 sections, every 1.5 µm). Fertilization cone was located at middle section of the cell (side view) or rather at the top of the cell (top view). Events of the fast migration phase are highlighted.

(h) Super-resolution (airyscan) images of zygotes fixed between 4 to 5 hours post insemination (hpi) and stained for F-actin with of Phalloidin-Alexa488 displayed at grayscale (upper panel) and as fluorescence intensity map (pseudocolour, 12-Bit grayscale) with the corresponding look-up table shown as a colour scale bar ranging from intensity levels 1 (low) to 4096 (high). Sorted according to their stage from onset

of pronuclear migration. Yellow arrowheads mark increased F-actin levels between cell cortex and male pronucleus.

(i) Histogram illustrating percentage of DMSO- or Nocodazole-treated zygotes with cortical mClover3-Spire2 enrichment in respect to the position of the forming female pronucleus quantified from Fig. 6g. Peripheral refers to pronuclei forming close to the zygote's surface, while inside refers to pronuclei forming distant from zygote's surface. Data are mean, with error bars displaying s.d. from four independent experiments from a total number of analysed zygotes (italics).

(j) Three-dimensional time-lapse images of the fertilization cone (brightfield, single z-section) and the male pronucleus (H2B-mCherry, z-projection of 7 sections, every 1.5 µm) in live zygotes expressing SNAP-Rab11a or SNAP-Rab11a^{S25N}. The two events during the fast migration phase are indicated.

(k) Box plot showing time from pronuclear migration until fertilization cone has completely flattened is measured from (j). Box plot showing median (line), mean (small square), 5th, 95th (whiskers) and 25th and 75th percentile (box) was calculated was calculated from the total number of analysed zygotes (italics) and pooled from three independent experiments. Two-tailed student's t-test was used to test for significance ($p=0.1$). Rab11a^{S25N} expressing zygotes, male pronuclei maintained contact to the plasma membrane for about twice as long. During these thirty minutes, the male pronucleus expanded rapidly about 4.3 µm in diameter (Supplementary Fig. 1k) translating into a cell-inwards directed push at a velocity of ~0.07 µm/min.

(l) Box plot showing time from pronuclear migration until the male pronucleus detaches from the cell surface is measured from (j). Box plot showing median (line), mean (small square), 5th, 95th (whiskers) and 25th and 75th percentile (box) was calculated from the total number of analysed zygotes (italics) pooled from three independent experiments. Two-tailed student's t-test was used to test for significance ($p=0.001$). We observed a mildly delayed flattening of the cone, which together with the nuclear expansion (Supplementary Fig. 4k) may explain why fast migration may not be fully blocked upon inhibition of Rab11a (Fig. 3d). Scale bars, 10 µm.

Supplementary Figure 5

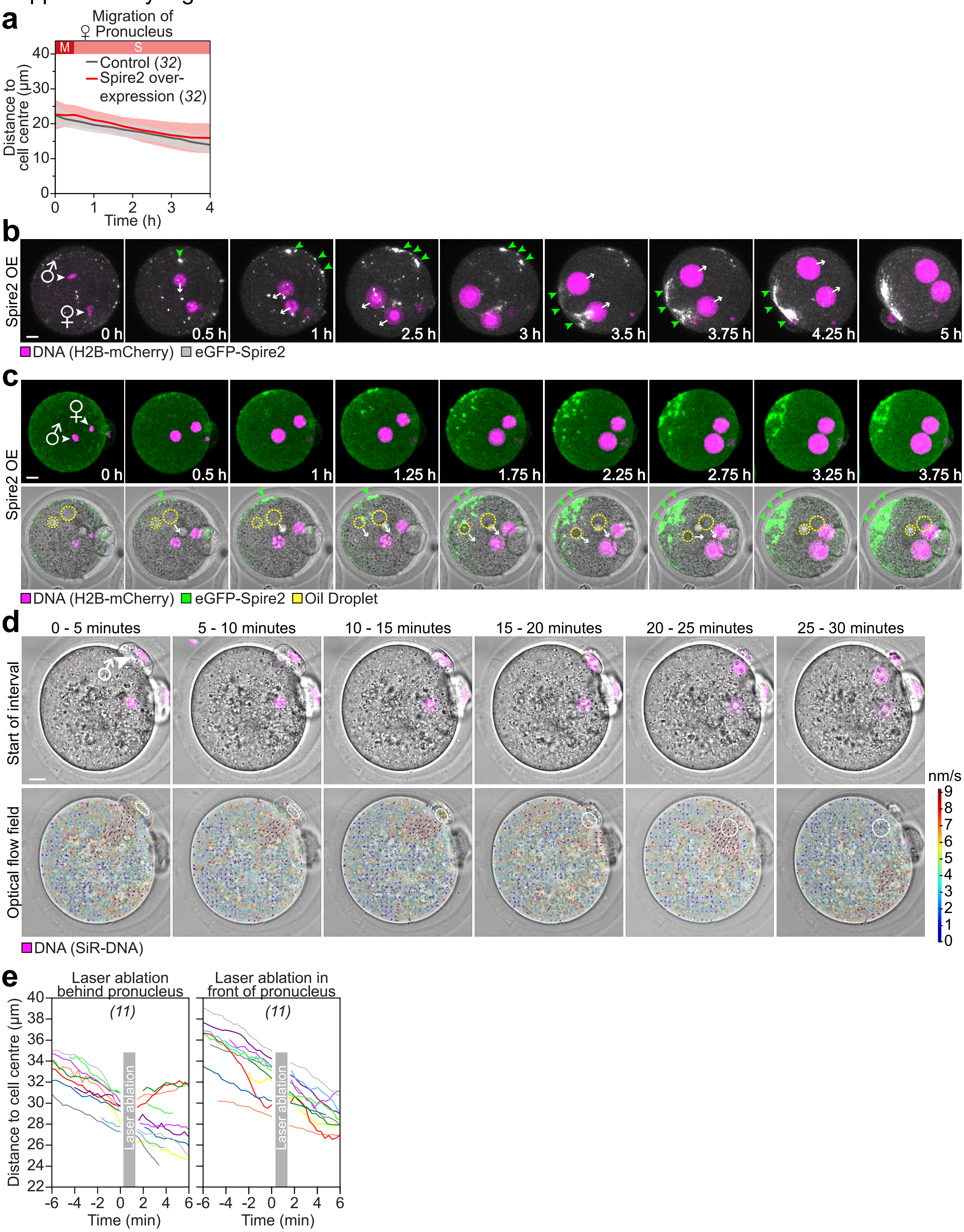

Supplementary Figure 5

(a) The mean distance (thick line) of female (♀) pronuclei centroids to zygote centre relative to pronuclear formation in zygotes overexpressing SNAP (control) or SNAP-Spire2 was calculated from data sets as shown in Fig. 5a. Phases of respective movement speeds are classified as in Fig. 1a. The total number of analysed zygotes (italics) was pooled from three independent experiments. S.d. shown as shaded areas.

(b) Three-dimensional time-lapse images of pronuclei (H2B-mCherry) in zygotes overexpressing (OE) high levels of EGFP-Spire2 represented as a maximum intensity projection (13 slices, every 5 µm). Female (♀) and male (♂) genomes are shown. Green arrowheads highlight the assembly of cortical Spire2 patches; white arrows indicate movement of pronuclei.

(c) Three-dimensional time-lapse images of pronuclei (H2B-mCherry) and oil droplets (yellow circles) in zygotes overexpressing high levels of EGFP-Spire2 represented as a maximum intensity projection (13 slices, every 5 µm). Bright-field images are presented as single z-projections. Female (♀) and male (♂) genomes are shown. Green arrowheads highlight the assembly of cortical Spire2 patches; white arrows indicate movement of oil droplets.

(d) Analysis of cytoplasmic streaming in early zygotes from time-point of pronuclear formation (0 minutes). Three-dimensional time-lapse images of pronuclei (SiR-DNA represented as maximum intensity projection (20 slices, every 2 um); bright-field images are presented as single z-slices. Male genome is shown. Optical flow field was determined within the defined interval as an average of optical flow fields calculated for each 10 seconds time-interval. The starting (dashed white line) and end position (continuous white line) of the male pronucleus within each defined interval are shown. The colour scale bar represents cytoplasmic flow vectors according to their speeds from 0 to 9 nm/s.

(e) The distance to the zygote's centre as individual tracks of pronuclei before and after laser ablation behind (left) or in front of (right) the male pronucleus was determined from data sets as in Fig. 5e. Total number of analysed zygotes specified in italics was pooled from three independent experiments. Scale Bars, 10um.

Supplementary Figure 6

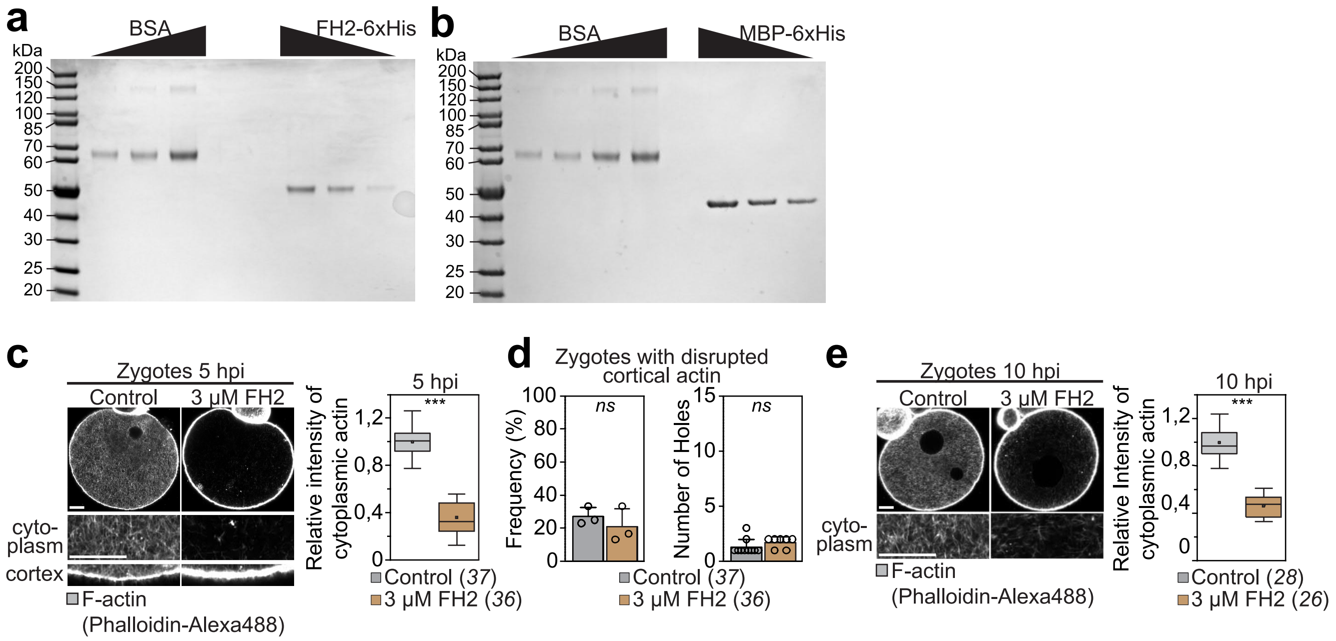

Supplementary Figure 6

(a, b) Coomassie-stained SDS-PAGE of purified 6xHis-tagged polypeptides FH2 (a) and MBP (b). For BSA reference (66kDa), increasing amounts of protein were loaded from left to right (80ng, 125ng and 250ng, and 500ng in (b) only). For FH2-6xHis (51 kDa) and MBP-6xHis (43 kDa), dilutions of the stock solution with decreasing concentrations were loaded from left to right (1:200, 1:400 and 1:800). The stock concentrations were calculated to 30 mg/mL for FH2-6xHis (a) and 110 mg/mL for MBP-6xHis (b). The protein ladder is labelled with the according sizes in kDa at the left of the gel.

(c) Left, super-resolution (airyscan) images of zygotes injected with 3μM MBP (control) or FH2, fixed 5 hours post insemination (hpi) and stained for F-actin with Phalloidin-Alexa488. A magnified region of the cytoplasmic actin network and the actin cortex are shown in the bottom panels. Right, box plot of the relative intensities of cytoplasmic actin were calculated from the mean intensity of Phalloidin-Alexa488 measured from a single section in the equatorial region of the zygote and normalized to the mean intensity measured for control cells per experiment. Box plot showing median (line), mean (small square), 5th, 95th (whiskers) and 25th and 75th percentile (box) was calculated from the total number of analysed zygotes (italics*)* pooled from three independent experiments. Two-tailed student's t-test was used to test for significance ($p<0.0001$). Scale bars, 10 μm.

(d) Left, the frequency of zygotes with disrupted cortical actin were calculated for zygotes injected with 3μM MBP (control) or FH2 from data sets as shown in (c). Data are mean with errors bars displaying s.d. from three independent experiments. Total number of analysed zygotes in italics. Two-tailed student's t-test was used to test for significance ($p=0.5$). Right, the mean number of holes per zygotes calculated as mean ± s.d. from all zygotes with disrupted cortical actin. Two-tailed student's t-test was used to test for significance ($p=0.2$).

(e) Left, super-resolution (airyscan) images of zygotes injected with 3μM MBP (control) or FH2, fixed 10 hpi and stained for F-actin with of Phalloidin-Alexa488. A magnified region of the cytoplasmic actin network in the bottom panels. Right, box plot of the relative intensities of cytoplasmic actin measured as described in (c). Box plot showing median (line), mean (small square), 5th, 95th (whiskers) and 25th and 75th percentile (box) was calculated from the total number of analysed zygotes (italics*)* pooled from

three independent experiments. Two-tailed student's t-test was used to test for significance ($p<0.0001$). Scale bars, 10 μm.

Supplementary Figure 7

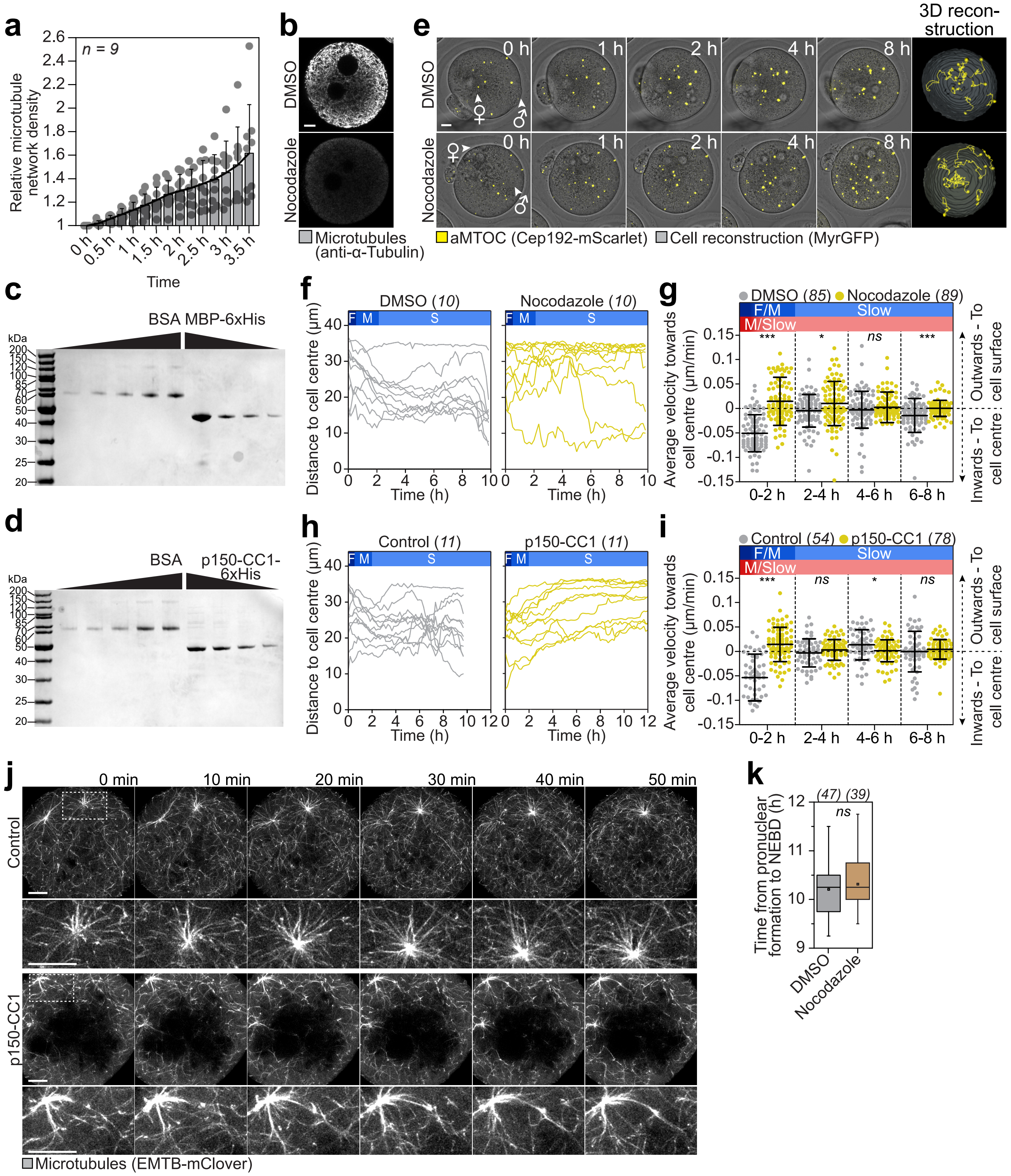

Supplementary Figure 7

(a) Histogram (mean ± s.d.) of relative density of the cytoplasmic microtubule network in zygote quantified from data sets as shown in Fig. 6a from a total number of analysed zygotes specified (italics) and pooled from three independent experiments.

(b) Images of zygotes treated with DMSO (control) or 10 µM Nocodazole, fixed 5 hours post insemination (hpi) and stained for microtubules (anti-α-tubulin). Displayed as z-projection of 3 sections (every 1 µm).

(c, d) Coomassie-stained SDS-PAGE of purified 6xHis-tagged polypeptides MBP (c) and p150-CC1 (d). For BSA reference (66kDa), increasing amounts of protein were loaded from left to right (50ng, 80ng, 125ng, 250ng, and 500ng. For MBP-His (43 kDa), dilutions of the stock solution with decreasing concentrations were loaded from left to right (1:20, 1:50, 1:100 and 1:200). For p150-CC1-His (41 kDa), dilutions of the stock solution with decreasing concentrations were loaded from left to right (1:50, 1:100, 1:200 and 1:400). The stock concentrations were calculated to 36 mg/mL for MBP-His (c) and 47 mg/mL for p150-CC1-His (d). The protein ladder is labelled with the according sizes in kDa at the left of the gel.

(e) Three-dimensional time-lapse images of acentriolar microtubule-organizing centres (aMTOCs) (Cep192-mScarlet, z-projection) and bright-field relative to pronuclear formation (0 h) in zygotes treated with DMSO or 10 µM Nocodazole. Female (♀) and male (♂) genomes are labelled. Three-dimensional iso-surface reconstruction of cell surface (MyrGFP) and tracking of aMTOCs during pronuclear stage.

(f) Graphs display tracks of aMTOCs relative to pronuclear formation (0 h) in respect to cell centre in DMSO- or Nocodazole-treated zygotes as calculated from data sets shown in (e). Phases of respective movement speeds as classified in Fig. 1a. The total number of analysed aMTOCs are specified in italics each from one representative cell per condition.

(g) Statistical plots of average velocities of aMTOCs during pronuclear migration in DMSO- or Nocodazole-treated zygotes calculated from (f). The total number of analysed aMTOCs are specified in italics each from 9 cells per condition and pooled from three independent experiments. Statistical plots show mean ± s.d. Two-tailed student's t-test was used to test for significance (left to right: $p<0.0001$, $p=0.01$, $p=0.3$ and $p=0.0006$).

(h) Graphs display tracks of aMTOCs relative to pronuclear formation (0 h) in respect to cell centre in zygotes micro-injected with 30 µM MBP (control) or p150-CC1. The total number of analysed aMTOCs are specified in italics each from one representative cell per condition.

(i) Statistical plots of average velocities of aMTOCs during pronuclear migration in zygotes micro-injected with 30 µM MBP (control) or p150-CC1 calculated from (h). The total number of analysed aMTOCs are specified in italics each from 7 or 9 cells per condition and pooled from three independent experiments. Statistical plots show mean ± s.d. Two-tailed student's t-test was used to test for significance (left to right: $p<0.0001$, $p=0.1$, $p=0.02$ and $p=0.5$).

(j) Three-dimensional super-resolution (airyscan) time-lapse images of microtubules (EMTB-mClover) in live zygotes micro-injected with 30 µM MBP (control) or p150-CC1 (z-projection of 3 confocal sections, every 1.5 µm). Time-point 0 marks the start of acquisition about 5.5 hpi. The regions outlined in the overviews are magnified in bottom panel.

(k) Box plot representing the time from pronuclear formation until NEBD in zygotes treated with DMSO or 10 µM Nocodazole quantified from data sets shown in Fig. 6b. Box plot showing median (line), mean (small square), 5th, 95th (whiskers) and 25th and 75th percentile (box) was calculated from a total number of analysed zygotes (italics) pooled from four independent experiments. Two-tailed student's t-test was used to test for significance ($p=0.3$). Scale bars, 10 µm.

Supplementary Figure 8

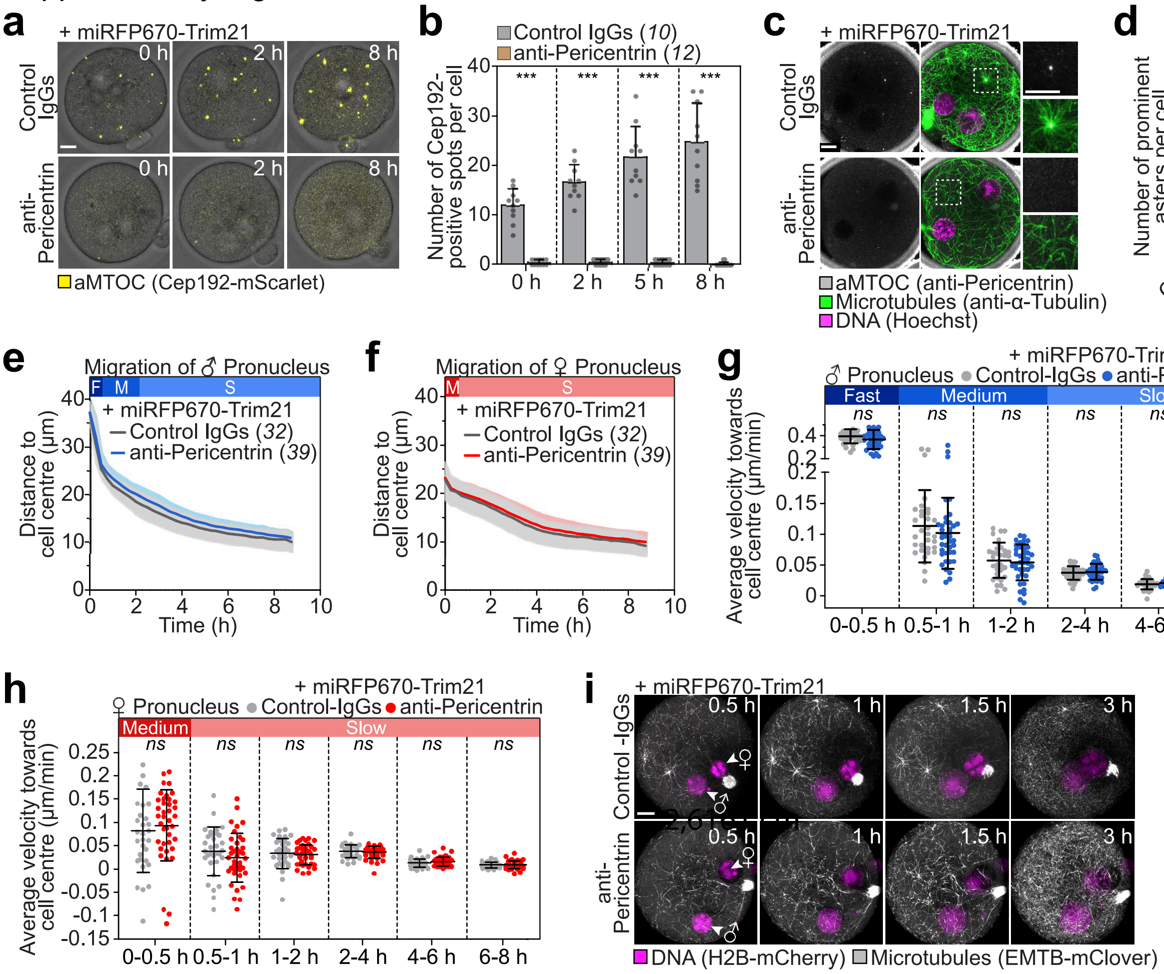

Supplementary Figure 8

(a) Three-dimensional time-lapse images of acentriolar microtubule-organizing centres (aMTOCs) (Cep192-mScarlet, z-projection) and bright-field relative to pronuclear formation (0 h) in zygotes expressing miRFP670-Trim21 and microinjected with Mouse-IgG (control) or Pericentrin-IgG.

(b) Histogram (mean ± s.d.) of number of Cep192-positive spots (aMTOCs) per zygote half quantified from data sets as shown in (a) from a total number of analysed zygotes specified (italics) and pooled from three independent experiments. Two-tailed student's t-test was used to test for significance (left to right: $p<0.0001$, $p<0.0001$, $p<0.0001$ and $p<0.0001$).

(c) Three-dimensional images of zygotes expressing miRFP670-Trim21 and microinjected with Mouse-IgG or Pericentrin-IgG, fixed 5 hpi and stained for aMTOCs (anti-pericentrin), microtubules (anti-α-tubulin) and DNA (Hoechst) (z-projection of 11 sections, every 1 µm). The regions outlined in the overviews are magnified in bottom panel.

(d) Histogram (mean ± s.d.) of the number of prominent microtubule asters per cell in zygotes expressing miRFP670-Trim21 and microinjected with Mouse-IgG or Pericentrin-IgG quantified from data sets as shown in (c). Total number of analysed zygotes (italics) and pooled from three independent experiments. Two-tailed student's t-test was used to test for significance ($p<0.0001$).

(e, f) The mean distance (thick line) of male (♂, (e)) and female (♀, (f)) pronuclei centroids to zygote centre relative to pronuclear formation (0 h) in zygotes expressing miRFP670-Trim21 and microinjected with Mouse-IgG or Pericentrin-IgG. Phases of respective movement speeds are classified as in Fig. 1a. The total number of analysed zygotes (italics) was pooled from three independent experiments. S.d. shown as shaded areas.

(g, h) Statistical plots of average velocities of male (♂, (g)) and female (♀, (h)) pronuclei during pronuclear migration in zygotes expressing miRFP670-Trim21 and microinjected with Mouse-IgG or Pericentrin-IgG calculated from (e) and (f) with the same sample sizes as in (e) and (f) respectively. Statistical plots show mean ± s.d. Two-tailed student's t-test was used to test for significance (left to right: (g) $p=0.2$, $p=0.4$, $p=0.6$, $p=0.5$, $p=0.2$ and $p=0.06$; $p=0.6$, $p=0.3$, $p=0.7$, $p=0.6$, $p=0.2$ and $p=0.7$).

(i) Three-dimensional super-resolution (airyscan) time-lapse images of microtubules (EMTB-mClover) and pronuclei (H2B-mCherry, magenta) relative to pronuclear

formation (0 h) in live zygotes expressing miRFP670-Trim21 and microinjected with Mouse-IgG or Pericentrin-IgG. Female (♀) and male (♂) genomes are labelled. Scale bar, 10 μm.

Supplementary Figure 9

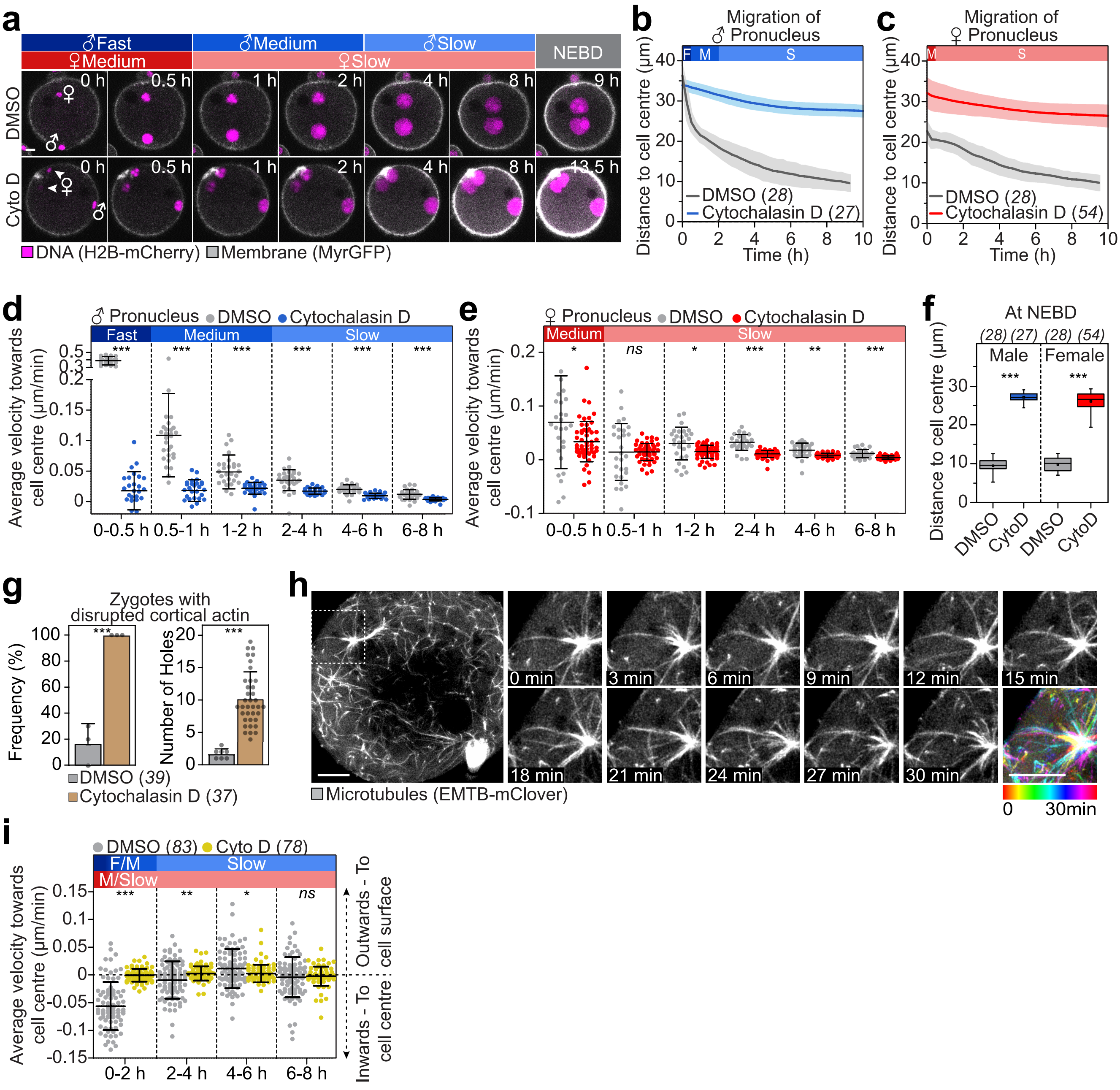

Supplementary Figure 9

(a) Three-dimensional time-lapse images of pronuclei (H2B-mCherry) and the cell surface (MyrGFP) relative to pronuclear formation (0 h) in zygotes treated with DMSO or 5 µg/mL Cytochalasin D until nuclear envelope breakdown (NEBD). Female (♀) and male (♂) genomes are labelled. Phases of respective movement speeds are classified as in Fig. 1a. Treatment with Cytochalasin D disrupted cytokinesis and resulted in formation of two female pronuclei.

(b, c) The mean distance (thick line) of male (♂, (b)) and female (♀, (c)) pronuclei centroids to zygote centre relative to pronuclear formation (0 h) in DMSO- or Cytochalasin D-treated zygotes was calculated from data sets as shown in (a). The total number of analysed zygotes (italics) was pooled from three independent experiments. S.d. shown as shaded areas.

(d, e) Statistical plots of average velocities of male (♂, (e)) and female (♀, (e)) pronuclei in DMSO- or Cytochalasin D-treated zygotes calculated from (b) and (c) with the same sample sizes as in (b) and (c). Statistical plots show mean ± s.d. Two-tailed student's t-test was used to test for significance (left to right: (d) $p<0.0001$, $p<0.0001$, $p<0.0001$, $p<0.0001$, $p<0.0001$ and $p<0.0001$; (e) $p=0.04$, $p=0.97$, $p=0.02$, $p<0.0001$, $p=0.001$ and $p<0.0001$).

(f) Box plot of distances of pronuclei centroids to zygote centre at NEBD in DMSO- or Cytochalasin D-treated zygotes calculated from data sets in (a). Box plot showing median (line), mean (small square), 5th, 95th (whiskers) and 25th and 75th percentile (box) was calculated from the total number of analysed zygotes (italics*)* pooled from three independent experiments. Two-tailed student's t-test was used to test for significance (male $p<0.0001$ and female $p<0.0001$).

(g) Left, the frequency of zygotes with disrupted cortical actin were calculated for zygotes treated with DMSO or Cytochalasin from data sets as shown in Fig. 7f. Data are mean with a total number of analysed zygotes in italics, with error bars displaying s.d. from three independent experiments. Two-tailed student's t-test was used to test for significance ($p=0.01$). Right, the mean number of holes per zygote calculated in zygotes with disrupted cortical actin. Two-tailed student's t-test was used to test for significance ($p<0.0001$).

(h) Super-resolution (airyscan) time-lapse images of microtubules (EMTB-mClover3, z-projection of 2 sections, every 1 µm) in live zygotes. The outlined region is magnified. Time-point 0 min marks the start of acquisition. Six time-points from 0 min to 30 min

(6 minutes-intervals) were superimposed using Image J's Temporal-Colour Coder and represented in pseudocolour with the corresponding look-up table shown as a colour scale bar ranging from time-point 0 to 30min.

(i) Statistical plots of average velocities of acentriolar microtubule-organising centres (aMTOCs) during pronuclear migration in zygotes treated with DMSO or Cytochalasin D and calculated from data sets shown in Fig. 6i. Phases of respective movement speeds are classified as in Fig. 1A. The total number of analysed aMTOCs are specified in italics each from 9 cells per condition and pooled from three independent experiments. Statistical plots show mean ± s.d. Two-tailed student's t-test was used to test for significance (left to right: $p<0.0001$, $p=0.003$, $p=0.04$ and $p=0.7$). Scale Bars, 10 µm.

Supplementary Figure 10

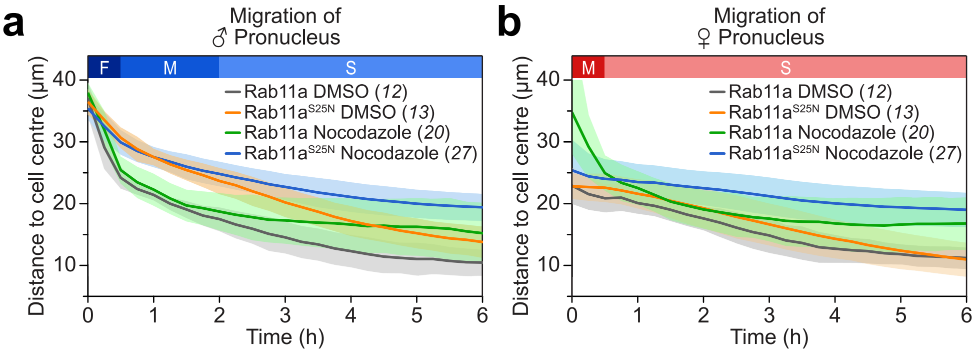

Supplementary Figure 10

(a, b) The mean distance (thick line) of male (♂, (a)) and female (♀, (b)) pronuclei centroids to zygote centre during pronuclear migration in zygotes expressing SNAP-Rab11a or SNAP-Rab11a^{S25N} treated with DMSO or 10 µM Nocodazole was calculated from data sets as shown in Fig. 9a. Phases of respective movement speeds as classified in Fig. 1a. Total number of analysed zygotes specified in italics was pooled from three independent experiments. S.d. shown as shaded areas.

Supplementary Figure 11

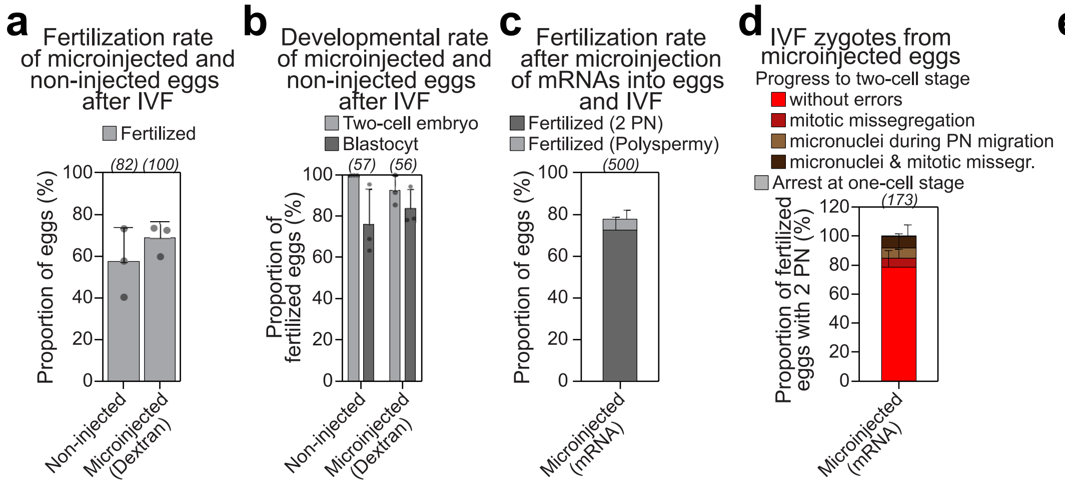

Supplementary Figure 11

(a) Histogram showing proportion of eggs that were in vitro fertilized (IVF). Prior to IVF, eggs were either non-injected or microinjected with Dextran. Fertilized eggs were evident from presence of pronuclei. Data are mean, with error bars displaying s.d. from three independent experiments. The total number of analysed zygotes is specified in italics.

(b) Histogram showing proportion of fertilized eggs from (a) that developed to 2-Cell (1 dpi) and blastocyst stage (4 dpi).

(c) Histogram showing proportion of eggs that were in vitro fertilized. Prior to IVF, eggs were microinjected with reporter mRNA encoding for H2B-mCherry and MyrGFP. Zygotes were categorized by their number of pronuclei: two pronuclei (PN) (normal fertilization) or three pronuclei (polyspermic fertilization). Data are mean, with error bars displaying s.d. from eleven independent experiments. The total number of analysed zygotes is specified in italics.

(d) Histogram showing frequency of errors observed in zygotes from (c). In zygotes that completed cleavage, errors were categorized into errors during pronuclear stage (namely micronuclei caused by loss of individual chromosomes from maternal genome prior to pronuclear formation) or mitotic stage (namely chromosome segregation errors including lagging or misaligned chromosomes that led to formation of micronuclei in the two-cell embryo). A low proportion of eggs arrested at one-cell stage either in pronuclear stage or during mitosis. Data are mean, with error bars displaying s.d. from eight independent experiments. The total number of analysed zygotes is specified in italics.

(e) Histogram showing proportion of in vivo fertilized eggs that developed to 2-Cell (1 dpi) and blastocyst stage (4 dpi). Data are mean, with error bars displaying s.d. from three independent experiments. The total number of analysed zygotes is specified in italics.

Supplementary Figure 12

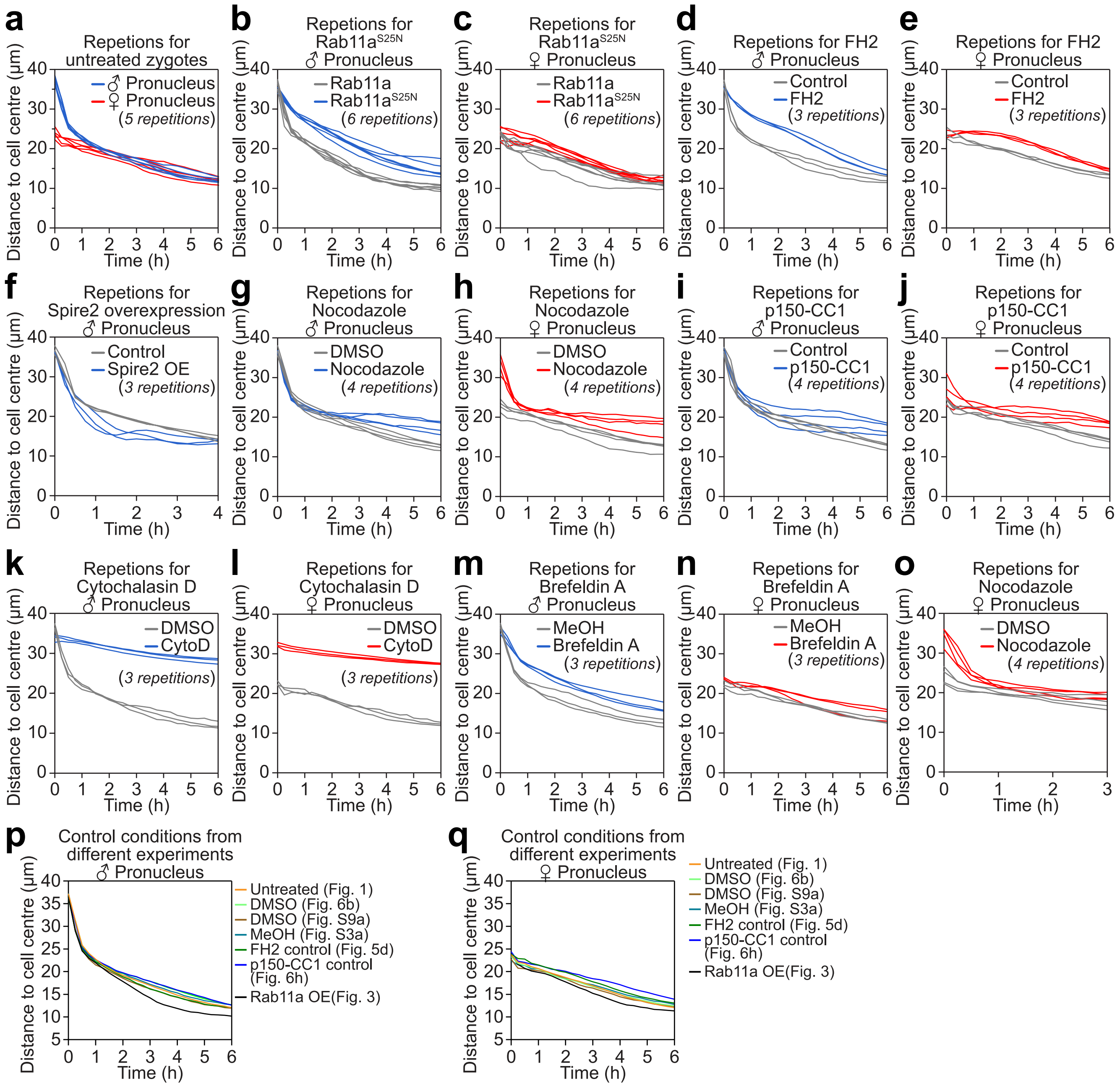

Supplementary Figure 12

(a-o) Mean distance to cell centre from independent repetitions for different experimental conditions were plotted over time.

(p-q) Mean distance to cell centre from separate experiments for different control conditions were plotted over time. Overall, the curves are very similar between different control groups. The male pronuclei in the Rab11a-overexpressing cells (p) move faster over a longer time-period, similar but milder to what we observed for Spire2 overexpression. This might be due to an enhancement of the actin-polymerization-dependent mechanism that pushes the pronuclei away from the zygote's surface. Also, the female pronucleus was slightly accelerated in Rab11a-overexpressing zygotes. The curves of the female pronuclei in the DMSO, Methanol (MeOH) and untreated control groups are highly consistent (q). In contrast, MBP microinjection (control for FH2 and p150-CC1 injection) caused a slight decrease in female pronuclear migration (q), however both experimental groups were again very consistent. This suggests that MBP injection has a minor decelerating effect on female pronuclear migration.

Supplementary Figure 13

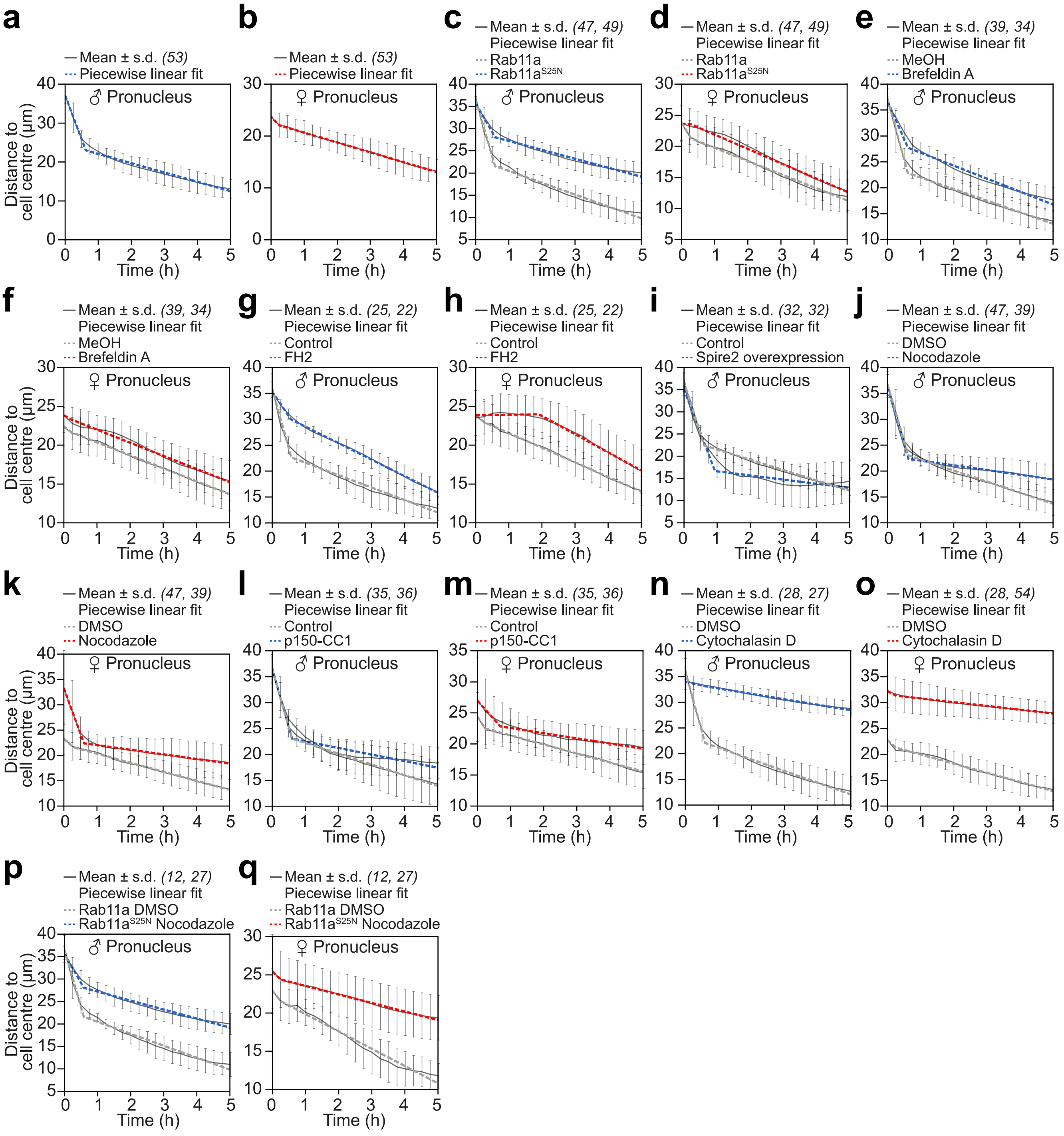

Supplementary Figure 13

(a-q) Mean (± s.d. shown as bars) distances to the cell centre were plotted for stated conditions as thin black line. Fitted curves (thick dashed lines) were created by piecewise linear regression after concatenation of data points (for details see Methods and Materials).

Supplementary Table 1

Group	Condition	Duration from … to … (hours): Pronuclear migration - NEBD	Duration from … to … (hours): NEBD - Anaphase onset	Duration from … to … (hours): NEBD - Cleavage	Frequency (%) *events (total N)*: Micronuclei during pronuclear migration [1]	Frequency (%) *events (total N)*: Arrest prior to NEBD	Frequency of errors in mitosis (%) *events (total N)*: Arrest at pro-/metaphase	Frequency of errors in mitosis (%) *events (total N)*: Chromosome missegregation [2]	Frequency of errors in mitosis (%) *events (total N)*: Failure to unite parental genomes	Disrupted cytokinesis Frequency (%) *events (total N)*	Time from NEBD to unification of parental genomes *(events)*
Inhibition of actin polymerization-dependent migration	Rab11a	9.9 ± 1.0 (Fig. S3i)	2.1 ± 0.3	2.3 ± 0.3	21 ± 15 *11 (47)*	0 *0 (47)*	3 ± 8 *1 (47)*	11 ± 12 *6 (47)*	0 *0 (47)*	0 *0 (46)*	0.9 ± 0.3 *(47)*
	Rab11a^{S25N}	10.0 ± 1.0 (n.s.)	2.0 ± 0.5 (n.s.)	2.8 ± 1.0 (**)	0 *0 (49)*	0 *0 (49)*	0 *0 (49)*	3 ± 8 *1 (49)*	0 *0 (49)*	12 ± 15 *6 (49)*	0.8 ± 0.3 (*) *(49)*
	Control	9.8 ± 0.7	2.1 ± 0.6	2.3 ± 0.6	3 ± 5 *1 (25)*	0 *0 (25)*	0 *0 (25)*	3 ± 5 *1 (25)*	0 *0 (25)*	0 *0 (25)*	0.9 ± 0.2 *(25)*
	FH2	10.0 ± 0.6 (n.s.)	2.1 ± 0.5 (n.s.)	2.4 ± 0.5 (n.s.)	0 *0 (22)*	0 *0 (22)*	0 *0 (16)*	4 ± 7 *1 (16)*	0 *0 (22)*	0 *0 (16)*	0.8 ± 0.2 (n.s.) *(22)*
	MeOH	9.8 ± 0.5	1.9 ± 0.6	2.2 ± 0.6	4 ± 4 *2 (39)*	0 *0 (39)*	0 *0 (39)*	6 ± 11 *3 (39)*	0 *0 (39)*	0 *0 (39)*	0.8 ± 0.2 *(39)*
	Brefeldin A	10.3 ± 0.5 (***)	2.0 ± 0.3 (n.s.)	2.9 ± 0.8 (***)	0 *0 (34)*	0 *0 (34)*	3 ± 4 *1 (34)*	8 ± 8 *3 (34)*	4 ± 7 *1 (34)*	30 ± 20 *9 (33)*	0.8 ± 0.3 (n.s.) *(34)*
Inhibition of microtubule-dependent migration	DMSO	10.2 ± 0.5 (Fig. S7k)	2.1 ± 1.0	2.4 ± 1.0	3 ± 6 *1 (47)*	0 *0 (47)*	0 *0 (47)*	5 ± 10 *4(47)*	0 *0 (47)*	0 *0 (47)*	n.d. [3]
	Nocodazole	10.3 ± 0.5 (n.s.)	n.d. [3,6]	n.d. [3,6]	30 ± 9 *11 (39)*	0 *0 (39)*	100 *39 (39)*	n.d. [3,6]	100 *39 (39)*	n.d. [3,6]	n.d. [3,6]
	Control	10.7 ± 0.8	2.0 ± 0.6	2.2 ± 0.3	2 ± 5 *1 (35)*	0 *0 (35)*	0 *0 (35)*	11 ± 12 *3 (35)*	0 *0 (35)*	0 *0 (35)*	n.d. [3]
	p150-CC1	12.7 ± 1.3 (***)	2.4 ± 0.7 (***)	3.1 ± 0.4 (***)	2 ± 4 *1 (36)*	4 ± 8 *1 (36)*	8 ± 9 *3 (35)*	54 ± 22 *19 (35)*	26 ± 16 *8 (35)*	20 ± 28 *4 (32)*	n.d. [3]
	Acute DMSO	n.d. [3,4]	2.1 ± 0.3	2.5 ± 0.6	17 ± 21 *5 (24)*	0 *0 (24)*	0 *0 (24)*	27 ± 15 *6 (24)*	0 *0 (24)*	0 *0 (24)*	1.1 ± 0.3 *(24)*
	Acute Cyto D	n.d. [3,4]	3.8 ± 1.5 (***)	n.d. [3,5]	28 ± 12 *8 (30)*	27 ± 31 *10 (30)*	18 ± 20 *3 (20)*	37 ± 25 *8 (20)*	9 ± 8 *2 (20)*	100 *17 (17)*	1.8 ± 0.8 (***) *(18)*
Inhibition of actin- & MT-dep. migration	DMSO	10.1 ± 0.5	2.4 ± 0.3	2.7 ± 0.4	3 ± 5 *1 (28)*	0 *0 (28)*	0 *0 (28)*	0 *0 (28)*	0 *0 (28)*	0 *0 (28)*	1.3 ± 0.2 *(28)*
	Cyto D	11.8 ± 1.5 (***)	2.1 ± 0.3 (n.s.)	n.d. [3,5]	0 *0 (27)*	9 ± 8 *3 (27)*	10 ± 14 *1 (13)*	51 ± 16 *7 (13)*	69 ± 26 *18 (24)*	100 *12 (12)*	2.1 ± 0.9 (n.s.) *(6)*
	Rab11a DMSO	9.8 ± 0.3	2.0 ± 0.6	2.2 ± 0.6	27 ± 35 *3 (12)*	0 *0 (12)*	0 *0 (10)*	21 ± 30 *3 (10)*	0 *0 (10)*	0 *0 (10)*	n.d. [3]
	Rab11a^{S25N} Nocodazole	9.7 ± 0.7 (n.s.)	n.d. [3,6]	n.d. [3,6]	3 ± 5 *1 (27)*	0 *0 (27)*	100 *27 (27)*	n.d. [3,6]	100 *27 (27)*	n.d. [3,6]	n.d. [3,6]

Supplementary Table 1

Table summarizing various parameters as mean ± s.d. (calculated from at least 3 independent repetitions) describing progression of mitosis in the one-cell embryos exposed to different manipulations during pronuclear migration and cell division. The times of pronuclear migration, anaphase onset and cleavage were determined from total number of zygotes as specified in italics in the following columns. For following parameters, number of observed events or total number of analysed zygotes as specified in italics and in brackets. Two-tailed student's t-test was used to test for significance.

Footnotes:

[1] Loss of single chromosomes during or after meiosis II (before pronuclear formation) resulting in one or several micronuclei.

[2] Misaligned or lagging chromosomes resulting in micronuclei in two-cell stage.

[3] Not determined.

[4] Addition of Cyto D after formation of pronuclei, thus individual zygotes could not be monitored from pronuclear formation to nuclear envelope breakdown (NEBD).

[5] Cytochalasin D disrupts cleavage in all zygotes.

[6] Nocodazole prevents spindle formation and zygotes arrest after NEBD with two paternal sets of chromosomes never segregated.

Supplementary Table 2

	Male pronuclei	Intersection point		Segment slopes (µm/min)		Female pronuclei	Intersection point		Segment slopes (µm/min)	
		x_3 (minutes)	y_3 (µm)	Fast	Slow		x_3 (minutes)	y_3 (µm)	Fast	Slow
	Untreated	37.5 ± 1.4	23.0 ± 0.2	0.38 ± 0.02	0.040 ± 0.001	Untreated	15.3 ± 4.8	22.1 ± 0.3	0.11 ± 0.03	0.032 ± 0.001
Vesicle-dependent actin nucleation	Rab11a	34.2 ± 1.2	22.3 ± 0.2	0.40 ± 0.02	0.050 ± 0.001	Rab11a	17.5 ± 4.9	21.2 ± 0.3	0.14 ± 0.04	0.035 ± 0.001
	Rab11a^{S25N}	34.2 *[1]	28.5 ± 0.2	0.20 ± 0.01	0.052 ± 0.001	Rab11a^{S25N}	17.5 *[1]	23.5 ± 0.2	0.01 ± 0.03	0.038 ± 0.001
	MeOH	36.8 ± 1.4	22.9 ± 0.2	0.39 ± 0.02	0.037 ± 0.001	MeOH	13.0 *[2]	21.7 ± 0.1	0.06 ± 0.03	0.028 ± 0.001
	BFA	36.8 *[1]	27.8 ± 0.2	0.24 ± 0.01	0.042 ± 0.001	BFA	13.0 *[1]	23.3 ± 0.2	0.04 ± 0.03	0.028 ± 0.001
	Control	34.6 ± 1.7	22.7 ± 0.3	0.38 ± 0.02	0.040 ± 0.002	Control	16.0 *[2]	22.9 ± 0.2	0.06 ± 0.03	0.031 ± 0.001
	FH2	34.6 *[1]	30.0 ± 0.2	0.16 ± 0.01	0.053 ± 0.001	FH2	114.7 ± 12.7	24.0 ± 0.4	-0.001 ± 0.006	0.039 ± 0.003
	Control	36.7 ± 1.3	22.4 ± 0.2	0.39 ± 0.02	0.039 ± 0.001					
	Spire2 OE	56.4 ± 3.1	16.7 ± 0.4	0.33 ± 0.02	0.016 ± 0.003					
Micro-tubules	DMSO	37.8 ± 1.4	23.0 ± 0.2	0.36 ± 0.02	0.036 ± 0.001	DMSO	16.6 ± 5.6	21.6 ± 0.3	0.10 ± 0.03	0.030 ± 0.001
	Nocodazole	37.8 *[1]	22.4 ± 0.2	0.36 ± 0.01	0.015 ± 0.001	Nocodazole	36.4 ± 2.4	22.4 ± 0.3	0.30 ± 0.03	0.015 ± 0.002
	Control	36.4 ± 2.3	23.0 ± 0.3	0.37 ± 0.03	0.035 ± 0.001	Control	13.9 *[2]	22.5 ± 0.2	0.14 ± 0.03	0.024 ± 0.001
	p150-CC1	36.4 *[1]	23.0 ± 0.2	0.38 ± 0.01	0.021 ± 0.001	p150-CC1	41.6 ± 8.8	22.9 ± 0.3	0.10 ± 0.02	0.014 ± 0.002
Actin and micro-tubules	DMSO	35.9 ± 1.7	22.1 ± 0.3	0.39 ± 0.02	0.038 ± 0.002	DMSO	15.0 *[2]	21.3 ± 0.2	0.10 ± 0.03	0.029 ± 0.001
	Cytochalasin D	35.9 *[1]	33.1 ± 0.1	0.026 ± 0.001	0.018 ± 0.001	Cytochalasin D	15.0 *[1]	31.3 ± 0.2	0.06 ± 0.01	0.012 ± 0.001
	Rab11a DMSO	35.0 ± 2.1	21.6 ± 0.4	0.43 ± 0.03	0.044 ± 0.002	Rab11a DMSO	14.9 *[2]	21.5 ± 0.3	0.09 ± 0.05	0.038 ± 0.002
	Rab11a^{S25N} Nocodazole	35.0 *[1]	28.0 ± 0.2	0.22 ± 0.01	0.033 ± 0.001	Rab11a^{S25N} Nocodazole	14.9 *[1]	24.4 ± 0.3	0.07 ± 0.04	0.019 ± 0.001

Supplementary Table 2

Table summarizing parameters of fitted curved obtained by piecewise linear regression from Supplementary Fig. 13 (for details see Methods and Materials) after concatenation of data points of stated conditions. Grey shaded values are different between control and experimental group.

Footnotes:

[1] Constraint of x_3 to maintain segment length between control and experimental group for slope comparison.

[2] Constraint of x_3 required to due to ambiguous fitting.

Supplementary Table 3

List of primers used in this study.

**Primer name**	**Primer sequence (5’ → 3’)**
Myr-GFP_Forward	GCTAGCATGGGCTGCATCAAGAGCAAGCGCAAGGACAACCTGAACGACGACGAGGCCGCCATGGTGAGCAAGGGCGAGGAGC
Myr-GFP_Reverse	GAATTCGAAGCTTGAGCTCG
mScarlet_Forward	GCTAGCGCCACCATGGTGAGCAAGGG
mScarlet_Reverse	GCATGGACGAGCTGTACAAGCTCGAG
mScarlet-Rab11a_Forward	GCTGTACAAGCTCGAGCTCAGCATGGGCACCCGCGACGAC
mScarlet-Rab11a_Reverse	CCGTCGACTGCAGAATTCGATTAGATGTTCTGACAGCACTGCACCTTTG
mClover-Spire2_Forward	GCTAGCGCCACCATGGTGAG
mClover-Spire2_Reverse	CTCGAGACTTGTACAGCTCGTCCATG
Formin2-GFP_Forward	AGATCTATGGGGAACCAGGATGGGAAGC
Formin2-GFP_Reverse	GCGGCCGCTTACTTGTACAGCTCGTCCATGC
GFP-UtrCH_Forward	ACCGGTGCCACCATGGTGAGCAAGGGCGAGGAGC
GFP-UtrCH_Reverse	GCGGCCGCTTAGTCTATGGTGACTTGCTGA
EMTB-mClover_Forward	GGATCCACCGGTCGCCACCATGGTGAGCAAGGGCGAGGAGC
EMTB-mClover_Reverse	GCGGCCGCTTTAGCATTTAGGTGACACTATAGAATAG
miRFP670-Trim21_Forward	ACCGGTATGGTAGCAGGTCATGCCTCTGG
miRFP670-Trim21_Reverse	AGATCTGCTCTCAAGCGCGGTGATCC
MBP_Forward	TCTAGAAATAATTTTGTTTAACTTTAAGAAGGAGATATACCATGAAAATCGAAGAAGGCAAACTGG
MBP_Reverse	CTCGAGACCCAGATTATTATTGTTATTG
FH2_Forward	TCTAGAAATAATTTTGTTTAACTTTAAGAAGGAGATATACCATGAAGCCTCTCTATTGGACAAG
FH2_Reverse	CTCGAGTGCGGCCAATTCGGATCCCGTTTTCATGCTTATCTTCGC
